# Supplementary material for: The community engagement and empowerment cycle: FAIRagro’s framework to foster cultural change towards FAIR RDM practices in agrosystem science and beyond
Source: J Integr Bioinform. 2026 May 18;22(4):20250049. doi: 10.1515/jib-2025-0049 (PMC13526452; doi:10.1515/jib-2025-0049)
Supplement: Supplementary file 1 [file j_jib-2025-0049_suppl_001.pdf]

**TITLE PAGE**

Author names (one full given name, second name, initials with full stop and without blank)

Anne Sennhenn (1), James M Anderson (1), Sophie Boße (2), Carsten Hoffmann (3), Oliver Kirchgessner (4), Manuela König (4), Benjamin Leroy (5), Birte Lindstädt (2), Elena Rey Mazón (6), Wahib Sahwan (3), Marcus Schmidt (3), Lea Sophie Singson (7), Nikolai Svoboda (3), Lucia Vedder (8), Ulrike Stahl (4)

Title

# The Community Engagement and Empowerment Cycle: FAIRagro's Framework to Foster Cultural Change towards FAIR RDM Practices in Agrosystem Science and Beyond

Author affiliations (department, institution, city, (if applicable, state abbreviated), country)

(1) Leibniz Institute for Agricultural Engineering and Bioeconomy (ATB), Central IT and Information Management, Potsdam, Germany
(2) ZB MED Information Centre for Life Sciences, Köln, Germany
(3) Leibniz Centre for Agricultural Landscape Research (ZALF), Computation and Data Service Platform (CDP), Müncheberg, Germany
(4) Julius Kühn-Institut (JKI), Germany
(5) Technical University of Munich (TUM), World Agricultural Systems Center - Hans Eisenmann-Forum für Agrarwissenschaften, Munich, Germany
(6) Leibniz Institute of Plant Genetics and Crop Plant Research (IPK), Gatersleben, Germany
(7) Leibniz Institute for Information Infrastructure (FIZ), Karlsruhe, Germany
(8) University of Bonn, Institute of Geodesy and Geoinformation (IGG) - Information Management, Bonn, Germany

Corresponding author: Full postal address and email address

Dr. Anne Sennhenn
Leibniz Institute for Agricultural Engineering and Bioeconomy
Max-Eyth-Allee 100, 14469 Potsdam, Germany
asennhenn@atb-potsdam.de

Running head (max. 10 words)

Word count (max. 3500)

Number of Tables and Figures (max. 8)
Eight (Tables: one and Figures: seven)

Number of Video(s) (recommended not more than 2)
None

Number of Supplementary Material/Supplementary Tables/Figures (no limit)
Three

Contribution(s) as first, last, senior author (if applicable)


## Abstract

Open Science is reshaping how research is conducted, yet the sustainable adoption of FAIR (Findable, Accessible, Interoperable, Reusable) research data management (RDM) requires more than technical infrastructures. In agrosystem sciences, heterogeneous data and diverse institutional contexts make cultural change particularly challenging. FAIRagro, the NFDI consortium for agrosystem sciences in Germany, developed the **Community Engagement and Empowerment Cycle** to address three persistent gaps in FAIR implementation: limited engagement, insufficient integration practice, and the lack of guidance.

The Cycle comprises four components—**Identify, Translate, Communicate and Anchor**—and operates across individual, institutional and global levels. This paper presents selected measures implemented within the Cycle and discusses their interplay and impact, including Helpdesk support, Use Cases, training activities and communication formats. These are examined in light of five core assumptions: infrastructure is necessary but insufficient; cultural change requires coordinated socio-technical interventions; connectors are essential for bridging domain science and RDM; shared values and trust are major preconditions; and that sustainable change depends on shared ownership and active responsibility.

The paper also reflects on structural challenges and the need for internationally aligned FAIR ecosystems. It outlines effective approaches to community involvement, highlights remaining obstacles and offers pathways for advancing FAIR RDM in agrosystem science and beyond.



Keywords (3-6 keywords):
**Culture Change** *(—)*, **Participatory approaches** *(9000119)*, **Stakeholder engagement** *(8d6ec0cb)*, **Community management** *(331459)*, **Research data management** *(—)*, **Open science** *(—)*,


We recommend compliance with the Mesh guidelines: https://www.ncbi.nlm.nih.gov/mesh/

# Introduction

## Open Science as a Paradigm Shift

Open Science has become a defining paradigm shift in how research is conducted, evaluated, and shared. It is not a single measure but an umbrella term encompassing openness, transparency, rigour, and replicability across the entire research process. Practices such as open access, open data, open code, open methodology, open peer review, and open educational resources expand beyond technical provision and are deeply embedded in the notion of good scientific practice—from careful study design and data collection to publication and reuse (Finger et al., 2024). Yet, openness alone is insufficient. The FAIR principles—Findable, Accessible, Interoperable, Reusable (Wilkinson et al., 2016)—provide the essential foundation to make data and other research products truly usable and impactful. Thus, data are the backbone of knowledge creation, innovation, and evidence-based decision-making.

## Relevance of Open Data in Agricultural Sciences

The agrosystem sciences, as part of the agricultural sciences, are highly data-intensive, spanning soil, plant, climate, biodiversity, and socio-economic data at multiple spatial and temporal scales. Already more than a decade ago, Rosenzweig et al. (2013) demonstrated within the *Agricultural Model Intercomparison and Improvement Project* (AgMIP) the value of harmonised and high-quality datasets for advancing agricultural modelling. AgMIP showed that only when diverse datasets are standardised, comparable, and openly exchangeable could multi-model ensembles produce robust insights into climate impacts, yield stability, and resource-use efficiency. This work also highlighted that model credibility and transferability depend critically on shared, well-documented datasets and transparent modelling protocols. Today the importance of open and FAIR data is further underscored by the growing number of international initiatives, for instance the establishment of the Common European Agricultural Data Space (CEADS) and the European Partnership "Agriculture of Data" (AgData). With the rapid progress of AI technologies and model-based decision support, harmonised and interoperable datasets have become even more essential, as they directly determine the quality, bias, and applicability of machine-learning models and predictive tools. The demand for open, high-quality, and interoperable datasets has therefore further increased, as they are essential to train, validate, and apply data-driven methods in agricultural research and innovation. All emphasise that agricultural data are key to addressing global challenges and ensuring innovation at scale (European Commission, 2023 (SRIA)). A later systematic review further demonstrated that FAIR data can substantially contribute to sustainable agricultural performance, although full implementation across the sector remains limited (Ali and Dahlhaus, 2022). In this way, open and FAIR agricultural data directly contribute to the United Nations Sustainable Development Goals (SDG 2 "Zero Hunger," SDG 12 "Responsible Consumption and Production," and SDG 13 "Climate Action") and support major policy frameworks including the European Green Deal and the Farm to Fork Strategy. They are indispensable for tackling interconnected challenges at the nexus of food security, climate change, biodiversity loss, and resource conservation (Ali and Dahlhaus, 2022).

## Status Quo and Perspectives of Research Data Management (RDM)

The research data management (RDM) landscape in Germany—much like the international situation—has long been characterised by heterogeneity and fragmentation. A multitude of institutions, authorities, and research initiatives and projects related to or relevant for agricultural science developed their own practices in relative isolation. Repositories relevant to agrosystem and agricultural sciences were poorly harmonised and rarely interconnected neither across disciplines nor on an international scale. Research institutions, governmental bodies, and individual projects frequently managed their data in isolated silos and developed their own research data infrastructures with narrow disciplinary focus. This resulted in limited harmonisation and interoperability as well as insufficient data discoverability, visibility and reuse. Closing these gaps will

requires concerted integration efforts nationally and internationally, ensuring that the German RDM landscape reflects—and can effectively support—the inherently global nature of agricultural research.

Legal barriers and constraints continue to limit open and collaborative research practices in agrosystem sciences. First, legal uncertainties—arising from heterogeneous international regulations on copyright, licensing, and data protection—create ambiguity for researchers working with multinational datasets. As Iaia (2025) shows, even the applicability of open licences such as CC BY varies across jurisdictions, as many datasets generated in Germany do not qualify for copyright protection, while analogous data in other countries may. Second, questions of data ownership remain unresolved in many research settings, particularly in collaborative or institutionally complex projects. Unclear responsibilities and rights hinder data sharing even when technical or organisational pathways exist. Third, researchers frequently perceive a competitive disadvantage when sharing data early or openly, fearing that others might publish first or capitalise on long-term datasets. Surveys confirm that such concerns contribute to a marked attitude–behaviour gap: although openness is valued in principle, it is not consistently practised (Senft et al., 2022).

This situation is further reinforced by prevailing journal practices. In most agricultural science journals, the submission of underlying research data is not mandatory; instead, authors are generally required to provide a Data Availability Statement. However, these statements are seldom verified and rarely guarantee long-term accessibility, persistence, or reusability of the underlying data. As a result, even published research often lacks the transparency and reproducibility needed to support robust open science practices. These soft practices continue at the institutional level. Nearly all major research institutions have adopted data policies recommending FAIR practices, yet these are usually framed as guidance rather than enforceable obligations. Compliance is rarely mandated; instead, adherence is encouraged through training opportunities, helpdesks, and advisory services. Rooted in the institutional culture of academic freedom (*Wissenschaftsfreiheit*) and self-governance in German research organisations, data sharing therefore remains largely voluntary and dependent on individual motivation, disciplinary norms, and available support. These factors reinforce existing practices and slow the cultural change necessary for systematic and sustainable FAIR RDM.

## Role of National and International Initiatives

To address the challenges outlined above, a range of national and international initiatives has emerged. In Germany, the National Research Data Infrastructure (NFDI), funded by the German Research Foundation (DFG) since 2020, aims to overcome fragmented practices and establish sustainable, interoperable research data infrastructures. Organised into domain-specific consortia, the NFDI combines technical development with efforts toward cultural change, fostering broad bottom-up community engagement (NFDI, 2024).

Within this framework, FAIRagro—funded since 2023—serves as the dedicated consortium for agrosystem sciences. An overview of FAIRagro’s conceptual foundations and technical architecture is provided in Specka et al. (2023). The consortium brings together major agricultural research institutions and covers scales from gene to region, spanning a wide range of data types (Fig. 1). Originating from a broad community initiative in which these institutions acted as co-applicants and participants from the outset, FAIRagro develops scalable and interoperable RDM solutions in close dialogue with its community. A central focus lies in building upon and further developing existing infrastructures of the partner institutions and integrating them into a federated research data ecosystem.

**<Figure 1>**

Given the inherently international nature of agricultural research, cooperation and collaboration have been integral to FAIRagro’s mission from the beginning. FAIRagro builds on—and contributes

to—international initiatives that shape global RDM practices, thereby strengthening harmonisation and interoperability. This includes for instance institutional cooperation with organisations such as Wageningen University & Research (WUR) and INRAE, as well as collaboration with global initiatives such as AgMIP. FAIRagro also plays an active role in the Agrischemas Working Group, which develops a schema.org- and Bioschemas based framework for describing agricultural research data. In the life sciences, substantial progress has been made through ELIXIR and its German Network for Bioinformatics Infrastructure (de.NBI). FAIRagro seeks not only to align with these developments but also to extend them—both with respect to infrastructure integration and through joint community-engagement activities, including training and capacity-building.

As part of the broader NFDI structure, FAIRagro increasingly collaborates with neighbouring consortia within and beyond the life sciences. These efforts include contributions to cross-consortial base services (Basisdienste) and participation in NFDI Section initiatives (Sektionen) that address challenges relevant across disciplinary boundaries, including *(Meta)data Terminologies, Provenience*, *Training and Education*, as well as *Ethical, Legal and Social Aspects*. Through NFDI-level coordination, FAIRagro is further working towards integration with the European Open Science Cloud (EOSC), thereby enhancing international interoperability and increasing the visibility of agricultural research data within the wider European Open Science landscape.

However, aligning infrastructures at national and international levels addresses only one dimension of the challenge. From the outset, FAIRagro has recognised that lasting impact depends equally on cultural change and has therefore placed community-driven engagement at the heart of its efforts.

## The Importance of Cultural Change

Cultural change in science is increasingly recognised as a multi-layered process requiring shifts in values, incentives, and routines. Nosek et al. (2015) emphasise that sustainable transformation emerges when individual behaviours, community norms, and institutional structures align toward openness and transparency. In this view, research data management is not a purely operational task but a social endeavour grounded in awareness, motivation, reward systems, and shared responsibility.

While the Pyramid provides a useful illustration, other conceptual models highlight complementary dimensions (see Supplementary Material 1). Munafò et al.'s (2017) "*Manifesto for Reproducible Science* "stresses systemic reforms in incentives, transparency, training, and infrastructure. Nosek and Bar-Anan's "*Scientific Utopia*" calls for realigning scholarly communication and evaluation practices. Community engagement frameworks (CDC; Arnstein, 1969) underline the role of participation and empowerment, while socio-technical transition models (Geels, 2002) show that cultural and technical change must co-evolve. Together, these frameworks suggest that infrastructure development alone is insufficient without broader cultural and institutional support.

Several structural and systemic challenges complicate the establishment of a culture that meaningfully supports FAIR research data management. Limited academic credit for RDM and the scarcity of stable positions for data professionals (OECD, 2020) weaken the visibility and perceived value of data-related work. The temporary nature of many research data infrastructures (Borgman, 2015), the predominantly project-based funding of RDM initiatives, and the slow and indirect realisation of economic benefits from FAIR practices (European Commission, 2019) further reduce incentives for long-term behavioural change. Collectively, these conditions shape attitudes and expectations within the research community, influencing what is valued, rewarded, and prioritised. As outlined in the previous sections cultural change is also shaped by legal and governance uncertainties—such as heterogeneous interpretations of copyright originality across EU member states (Iaia, 2025)—which create ambiguity and risk aversion. At the same time, emerging concerns regarding benefit distribution influence researchers' willingness to engage openly: datasets curated

with substantial effort may later be reused by commercial actors (e.g. in AI development), raising questions of equity, attribution, and trust.

Taken together, these factors constitute the cultural environment in which researchers make decisions about data sharing. Empirical evidence shows that such social and cultural conditions—rather than technical availability alone—shape norms, expectations, and perceptions of fairness (Finger et al., 2024), ultimately determining whether FAIR practices become collectively embraced or remain optional and peripheral.

## The scope of this study

Implementing FAIR research data management (RDM) in agrosystem sciences remains challenging. As outlined in the previous sections, these challenges arise across three interconnected levels:
(1) **the individual level**, where skills, motivation, and norms shape daily practices;
(2) **the institutional level**, where policies, incentives, and governance structures determine what is supported and rewarded; and
(3) **the systemic or global level**, where standards, interoperability, and international collaboration condition the broader research ecosystem.

Addressing FAIR implementation effectively requires approaches that engage with all three dimensions simultaneously. This study therefore introduces the Community Engagement and Empowerment Cycle, a socio-technical framework that brings together infrastructural development with coordinated measures for capacity building, communication, and institutional anchoring. Its core premise is that meaningful progress in implementing FAIR practices depends on placing researchers and users at the centre of developments and treating FAIR RDM as a collaborative, iterative process rather than a purely technical one. FAIRagro has adopted this perspective by emphasising co-creation, participatory engagement, and continuous knowledge exchange, recognising that technical services only gain impact when they are understood, adopted, and embedded in real research workflows. As Medha Devare (Consultative Group on International Agricultural Research (CGIAR); FAIRagro Community Advisory Board) noted: *"Technical developments are straightforward; changing habits and culture is hard work.*"

The Community Engagement and Empowerment Cycle was designed to address this challenge. This paper traces its development and examines how it is implemented and put into practice within FAIRagro. It provides insights into how the different components of the Cycle are operationalised through concrete measures and activities, and how these, in combination, support the uptake of FAIR practices and contribute to cultural change in agrosystem sciences. The Cycle thus offers a structured framework that links infrastructure and service development with community engagement and empowerment, translating principles of cultural change into actionable components and providing a pathway for embedding FAIR practices sustainably across the field.

**The analysis is guided by the following core assumptions:**

**1. (Technical) Infrastructure is important but never sufficient:** FAIR adoption cannot be achieved by infrastructure alone. Even the most advanced services remain ineffective if they are not co-created with users, embedded in real research workflows, and actively supported through continuous engagement, usability improvement, and contextualised guidance.

**2. Cultural change is a socio-technical challenge; single interventions are insufficient to address its complexity:** Measures such as Use Cases, training, and helpdesk support unfold their full potential only when they are conceived as part of an overarching strategy that operates across all levels—individual, institutional, and systemic—and when they reinforce one another through synergies.

**3. Effectiveness of change depends on strong connections between institutions, initiatives, and communities:** FAIR adoption requires more than broadcasting information—it relies on active connectors who translate, contextualise, and circulate knowledge across institutional, disciplinary, and project boundaries, including the translation of technical developments from infrastructure and service providers to potential adopters and users.

**4. Shared values and trust are prerequisite socio-cultural conditions for change:** Common understanding and a shared mission create the foundational environment in which trust can grow, trusted intermediaries can take effect, and behavioural change becomes possible.

**5. Sustainable change requires shared ownership and active responsibility:** FAIR data and practices are not a service delivered to users, but a collective mission that must be carried across all levels—including individuals who shape daily practices, projects and initiatives that coordinate and advance these practices, and institutions that define the enabling environment for sustained implementation.

## Materials and methods - Developing the Community Engagement and Empowerment Cycle

### FAIRagro's Initial Concept (drawn from original proposal)

FAIRagro's initial concept built on a comprehensive needs assessment of the agrosystem science community. In preparation for the proposal, the first community survey was conducted in 2020 (Senft et al., 2022). The results presented in the following section clarified the key challenges, barriers, and needs expressed by the community. These insights informed the design of FAIRagro's overall structure. They were incorporated into the Task Areas (TAs, defined as the main areas of effort), ensuring that community needs were addressed directly in the consortium's planned measures and activities (Fig. 2).

Key findings and responses from the pre-Fairagro Community Survey are:

- **Use cases for community-driven implementation (Task Area 1 – Use Case Implementation):** Community needs identified in the pre-survey were systematically addressed through FAIRagro's task areas and Use Cases. With Use Case being a project addressing RDI challenges with a clear agrosystems research question/ context.
- **Guidance and support:** The community expressed a strong need for domain-specific FAIR guidelines, practical how-tos, and actionable checklists to support FAIR RDM and service implementation. FAIRagro therefore embedded these elements into its **Data Steward Service Center (DSSC)**, providing Helpdesk support as part of **Task Area 2 (Community Involvement and Networking)**.
- **Training and capacity building:** There was a clear demand for training. Early-career researchers requested introductory RDM courses, while experts sought advanced workshops with focus on technical implementation of FAIR principles. Training and community education were consequently defined as **core activities within Task Area 2**.
- **Metadata harmonisation:** Around 60% of respondents identified the lack of consistent metadata as a major barrier to data reuse. FAIRagro addressed this challenge in **Task Area 3 (Standardisation, Interoperability, Quality)** with dedicated activities on mapping and harmonising metadata schemas.
- **Data accessibility:** Many researchers reported that relevant datasets were difficult to find or not accessible. In response, FAIRagro planned the development of the FAIRagro Portal with a **Search Hub** and

the interconnection of existing RDIs through **advanced middleware** (as part of **Task Area 4 – Infrastructure Services**).

- **Integration into workflows:**
  Over 40% of respondents reported that existing RDM tools were poorly integrated into their research workflows. FAIRagro therefore prioritised the development of **tools and services** (e.g. SciWin, data quality tool) aligned with common analysis environments (**Task Areas 3 & 4**).
- **Legal and ethical clarity:**
  Uncertainties regarding data protection and licensing were identified as key barriers to data sharing. To address these, FAIRagro initiated dedicated activities on **legal frameworks and practical guidance** across **Task Areas 2, 3 and 4**.

**<Figure 2>**

The concept was designed as community-led, cross-institutional, and focused on interoperability and scalability (Fig. 2). At its core, "Use Case implementation" and "Community involvement and networking" provided the foundation for community-driven development. This included the coordination of flagship Use Cases, the launch and onboarding of new Use Cases, as well as communication and dissemination activities, systematic needs assessments, and the establishment of the Community Advisory Board (CAB). Training and education measures, together with the implementation of the DSSC, further supported capacity building and knowledge transfer. From the outset, the Use Cases and the DSSC were envisioned as the primary connecting elements between FAIRagro and its community.

## From Concept to Practice: Identifying Gaps

In practice, however, the initial approach revealed limitations. While advanced infrastructures and services were developed, still they often evolved in relative separation from the community, with limited user focus, insufficient institutional ownership, and only modest reach into partner institutions. This imbalance highlighted a fundamental challenge: technical solutions alone could not ensure meaningful adoption of FAIR practices.

To systematically assess progress and address these shortcomings, FAIRagro established the Community Advisory Board (CAB), an independent body of international experts in agrosystem sciences, data science, RDM, and related societal and political sectors. The CAB was tasked with providing strategic guidance, evaluating progress, issuing recommendations, and fostering connections with national and international infrastructures (see Supplementary Material 2).

CAB evaluations (2024, 2025) revealed several shortcomings:

- **Engagement gap**: Limited community involvement in co-creation and in the continuous improvement and further development of services, with feedback channels confined to a few Use Cases.
- **Integration gap**: Insufficient embedding of developed RDM services in research practice and a lack of adequate and deeper institutional integration.
- **Guidance gap:** Missing practical, domain-specific FAIR guidance and accessible entry points, leaving the community without tangible support.

Moreover, many of FAIRagro's early engagement formats primarily attracted already motivated or FAIR-inclined researchers, while more hesitant or sceptical community members remained largely absent from participatory feedback processes and co-creation activities.

The CAB further emphasised the need for clearer onboarding processes, stronger roles for ambassadors and data stewards as cultural change agents, more visible institutional commitments, and more effective mechanisms to demonstrate the adoption and implementation of infrastructures and services as part of tangible success stories (see Supplementary Material for a summary of CAB

reports). These findings were echoed at the first FAIRagro Community Summit (2024), where participants called for clearer and more accessible, and target-group-specific content.

In sum, while the initial infrastructure planning was solid, it proved insufficient without mechanisms to secure continuous participation, trust, and ownership. The identified gaps underscored that infrastructure can only succeed when coupled with meaningful service – community integration and cultural change.

## Development of the Community Engagement and Empowerment Cycle

The Community Engagement and Empowerment Cycle (Fig. 3) was developed as a systematic response to the engagement, integration and guidance gaps identified by the CAB and community feedback. While FAIRagro's initial measures provided important building blocks, they often remained fragmented and their impact limited when not embedded into a broader holistic approach. The Community Engagement and Empowerment Cycle therefore links technical dimensions with cultural and user perspectives, ensuring that services are not only developed but also understood, adopted, and sustained within everyday research practice in agrosystem science and beyond.
The further development of the Community Engagement and Empowerment Cycle placed particular emphasis on three critical insights.

- **Needs must be actionable**: Identifying community needs alone is insufficient if they are not translated into requirements in a form that developers can effectively act upon.
- **Solutions must be tangible**: Cutting-edge FAIR solutions, infrastructures and services remain underutilized if they are not made understandable, applicable, and directly relevant to researchers' daily practices.
- **Communication must be embedded**: Outreach has impact only when it reaches target groups within their actual work environments and disciplinary "bubbles", fostering authentic connections that can anchor and sustain FAIR practices.

**<Figure 3>**

Against this background, the Cycle provides a structured approach with four interconnected components: Understand, Translate, Communicate, Anchor, further illustrated below (Fig. 3).

Rather than a static framework, the Cycle represents a participatory and adaptive approach, aligned with established theories of Open Science and cultural change (Nosek et al., 2015; Munafò et al., 2017). This theoretical grounding ensures methodological flexibility while providing a structured lens through which the four dimensions — or consecutive components — are operationalised. At its heart lies the community: working not for the community but with the community. Accordingly, the Cycle is not limited to the development of infrastructures and services but extends to engaging stakeholders across disciplines and institutions, empowering researchers and organisations to actively shape and sustain FAIR practices.

To illustrate how the Cycle unfolds in practice we developed concrete measures and activities within each component and highlighted their interplay, achievements, impacts, and lessons learned. This analysis builds on perspectives and experiences gained from FAIRagro.

# Results - Implementing the Community Engagement and Empowerment Cycle

## Key Stakeholder and Main Target Group Analysis

FAIRagro engages with a broad spectrum of stakeholders who influence or are affected by its activities. Primary key stakeholders—those at the centre of FAIRagro's development efforts and the main beneficiaries of its services—include universities and universities of applied sciences, non-university research institutions, and governmental research institutes conducting agricultural research. In addition, infrastructures and service providers such as repositories, libraries, and data centres, which offer essential RDM services, form part of this core group. Professional societies, associations, and scientific networks, including NFDI consortia with links to agricultural sciences, also play a pivotal role as primary partners in shaping and disseminating FAIRagro's developments.

Secondary key stakeholders—positioned more at the periphery of FAIRagro's immediate development focus but nevertheless essential for the broader sustainability and long-term success of FAIRagro's mission—include governmental authorities, industry partners and small and medium-sized enterprises, private service providers, political institutions, funding agencies, and the wider public. While they are not the primary target of FAIRagro's technical and community measures, their regulatory influence, innovation potential, resource structures, and societal expectations critically shape the conditions under which FAIR practices can be adopted, scaled, and sustained over time.

From this environment, FAIRagro focuses on three main target groups:

**Users & Learners** – (young) agricultural scientists (PhDs, postdocs) as primary data producers and re-users. Their early adoption of FAIR practices is crucial for embedding cultural change into future research routines.

**Multipliers & Decision Makers** – senior scientists, project leaders, professors, and policy/funding actors. They hold significant influence, shaping adoption both within institutions and at the policy level, and act as multipliers to scale FAIR principles.

**Developers & Experts** – RDM specialists and technical professionals at infrastructures, libraries, service providers, and computing centres, as well as within research institutions. They design, build, and further develop infrastructure services, but are also involved in advancing standards and interoperability.

Together, these groups represent an estimated 32,000 individuals in Germany. Their differentiated roles—from producing and reusing data to developing and enabling infrastructures and services up to shaping policy—make them essential for FAIRagro's mission.

## Component 1: Identify and assess community needs

A central element of FAIRagro's strategy is the systematic identification and assessment of community needs. This process includes structured engagement with researchers, systematic evidence collection, the identification of concrete requirements, and a targeted analysis of existing gaps. The resulting evidence base provides the foundation for prioritisation and for the responsive development of services and infrastructures.

- **Community surveys** are a cornerstone. The 2023 survey on data quality (321 participants) revealed challenges in data discovery (70% of respondents), high demand for a central agricultural data search service, and calls for domain-specific FAIR guidelines and harmonised

metadata schemas (Uhlott et al., 2024). The 2024 survey on legal barriers (191 participants, 78 full responses) highlighted widespread legal uncertainty, frequent overestimation of license restrictions (e.g. CC BY), and demonstrated how legal concerns directly limit data publication. A third survey, in preparation, will focus on FAIRagro services to refine the portfolio and align product development with user expectations.
*Strengths:* high participation, actionable evidence, clear signals on community needs.
*Limitations:* rapid demand evolution (e.g. emerging AI technology and trends), uneven coverage of disciplines.

- **Use Case calls and applications** also provide systematic insights. In this context, a Use Case refers to a focused, domain-specific project that addresses a concrete RDI challenge in an agricultural research setting. The first Use Case call (2023/2024) attracted 12 applications, the second (2025) 16. Topics included data findability, metadata adoption, fragmented infrastructures, and legal uncertainties. Beyond these general patterns, Use Case applications offer in-depth perspectives on pressing RDM challenges and articulate concrete requirements, ranging from metadata harmonization and workflow integration to legal frameworks for sensitive data and interoperability across repositories (Sennhenn et al. 2025a, b). They capture needs from a broad spectrum of institutions and disciplines, thereby pointing directly to priorities for FAIRagro's infrastructure and service development (Tab. 1; Sennhenn et al., 2025). Descriptions of the 6 flag ship or initial Use Cases and the approved Use Cases are available at https://fairagro.net/community/use-cases/.
  *Strengths:* integrates new and emerging community demands; reveals concrete, up-to-date RDM needs
  *Limitations:* low funding rate (<20%); several excellent applications could not be supported

**<Table 1>**

- **Helpdesk requests** (>170 analysed from March 2023 to November 2025) provide additional evidence of real-world challenges. The three most frequent topics were policy, law and ethics, data publication and project support, followed by access to experts, data search and education (Fig. 4). This confirms persistent challenges around legal uncertainty and data publication and discovery, while also highlighting the need for guidance and training. A local cluster close to the coordinating institution of the FAIRagro Helpdesk in Brandenburg (Fig. 5) underlines our experience that RDM support needs to be not only domain-specific but profits from personal encounters. A majority of the requests so far have come from researchers whom the data stewards have met in person during training or at conferences. The map also shows that some regions, such as Schleswig-Holstein and Thuringia, have not yet generated requests, which likely reflects a lower density of agricultural research institutions rather than a lack of interest.
  *Strengths:* captures real, practice-based challenges across domains, highlights persistent barriers (legal uncertainty, publication, discovery)
  *Limitations:* strong bias toward users with prior personal contact, not fully representative of long-term structural needs

**<Figure 4>**

**<Figure 5>**

- **Event-based feedback** from workshops, training sessions, and conference exhibitions further underscores gaps in awareness of FAIR principles and RDM practices, particularly outside FAIRagro's core institutions.
  *Strengths:* Provides immediate, practice-based insights, reveals awareness gaps across diverse events and audiences
  *Limitations:* Feedback is opportunistic and not systematically sampled, overrepresents institutions attending FAIRagro-linked events

**Interplay & Impact:** The different measures are essential to capture the heterogeneity and diversity of the community and the broad range of stakeholders involved. They operate at varying levels of depth: Helpdesk tickets and event feedback provide punctual insights into community needs, surveys deliver structured analyses on specific focus topics, and Use Case calls and applications allow for in-depth examination of RDM challenges within particular research domains. Together, these measures provide representative insights into community needs and RDM challenges, helping to identify gaps and highlight opportunities, and thereby establishing a solid foundation for community-driven developments. Within the Cycle, they form the basis for the subsequent components, to which they also actively contribute: collecting needs marks the first step, followed by their systematic translation into RDM requirements through further activities and collaboration with additional actors.

## Component 2: Translate community needs into RDM requirements – and vice versa – FAIR solution (infrastructure and services) back into applicable practice.

Translation between community needs and RDM solutions lies at the heart of FAIRagro's participatory approach. While technical developments are often straightforward, understanding, prioritising, and integrating community requirements is a task that is frequently underestimated. Ensuring co-creation in practice, and aligning FAIR products, services, and tools with user perspectives and practical applicability, requires continuous mediation and transparent balancing of needs. Translation is therefore both a technical and a cultural process, shaping FAIRagro's strategic orientation as well as developments at the operational level.

**Translation in FAIRagro operates in two directions:**

- **Community needs → RDM requirements**: community needs must be systematically captured and translated into actionable RDM requirements.
- **RDM solutions → community practice**: existing or emerging FAIRagro solutions must be translated back into tangible and implementable practices.

### *FAIRagro Use Cases: Co-Creation in Agricultural Research*

Use Cases (UCs) are central instruments for translation. Their onboarding process mediates between FAIRagro's Task Areas and the scientific community, systematically capturing requirements and aligning them with ongoing developments. This mediation creates continuous feedback loops: UCs articulate requirements, TAs adapt services and standards, and outcomes are tested and re-integrated into research workflows of the UCs (Fig. 6). The mediation process favours the establishment of TA x UC working groups, where agricultural researchers work together with RDM experts e.g. on topics like metadata harmonisation and mapping, data interaction workflows from heterogeneous data sources and FAIR-compliant publication formats.

**<Figure 6>**

Concrete examples include TA x UC working groups on metadata harmonisation, crop model validation workflows, and Annotated Research Context (ARC) implementation. These groups

translate Use Case requirements into technical specifications — such as aligning BonaRes Longterm experiment (LTE) with ICASA dictionaries, extending MIAPPE for sensor-based field data, or developing so called *extract transform load* (ETL) pipelines for crop modelling — and thereby directly influence metadata standards, workflow automation, and infrastructure priorities. The FAIRagro interactive Use Case Map (https://fairagro.github.io/uc_coordination/, see Supplementary Material 3).

*Strengths:* structured co-creation between researchers and RDM experts, strong links and continuous feedback loops between UCs and TAs, direct translation of domain needs into technical specifications and standards
*Limitations:* collaboration intensity varies across UCs, requires sustained coordination and staff capacity, technical outcomes may outpace broader community adoption, depth of co-creation is limited when UCs lack internal RDM expertise

### *The FAIRagro Helpdesk: A Direct Link to the Community*

The **FAIRagro Helpdesk** (fairagro.net/helpdesk )is one central component of the FAIRagro Data Steward Service Center (DSSC). The DSSC is staffed by six Data Stewards with complementary expertise in plant breeding, precision farming, law and ethics, geodata, and data publication. In addition to operating the Helpdesk, the Data Stewards develop guidelines and training materials, support Use Cases, contribute to metadata harmonisation, and provide community-facing RDM consultancy. Within this broader portfolio, the Helpdesk offers personalised, context-aware support across the entire data lifecycle. Requests arrive via email, the online form, or personal encounters at community events, and are often handled collaboratively to ensure tailored solutions.

Beyond solving individual problems, the Helpdesk plays a strategic role in **needs assessment and prioritisation**. Tickets provide insights into recurring challenges, which are systematically analysed to guide FAIRagro's development priorities and strategic focus. Importantly, Helpdesk interactions do not only result in problem-solving; they also produce **FAIR(agro)-enriched outputs** that extend well beyond individual cases, three of which are:

- **A list of repository recommendations** (Rey-Mazón et al., 2024): developed in response to recurring questions about where to publish data; helps researchers navigate the repository landscape.
- **A roadmap to FAIR code publication** (Beyer et al., 2025): produced to meet increasing demand for guidance on code publication; downloaded >300 times and providing a legally sound, best-practice framework.
- **Metadata in agricultural practice** (Boße et al., 2025): a recently developed decision tree tool guides researchers step by step through the process of data documentation, helping them to handle metadata and standards for their specific research context.

Furthermore, FAIR(-agro)–enriched content and resources are collected, structured, and orchestrated within the developing **FAIRagro Knowledge Base** (knowledgebase.fairagro.net). This curated resource serves both as an internal reference for Data Stewards and as a public point of access for researchers, and it is interlinked with knowledge bases from related NFDI consortia to ensure cross-domain interoperability.

The Helpdesk thus represents a strong human link to the community. Data Stewards, with expertise in both research data management and agricultural science, effectively "speak both languages" and act as a crucial interface between technical solutions and domain practice. By taking responsibility for translation work, they ensure that individual requests evolve into tangible, reusable resources that not only solve individual problems but also feed into subsequent communication and support activities. This translation work will become increasingly important as more services are developed

and released. Accordingly, Data Stewards will need to expand their role towards acting as **service stewards** taking responsibility for making services implementable for the community, developing target groups–specific resources, and integrating these into their support and consultation activities.

*Strengths:* direct and personalised translation of community needs into actionable RDM requirements, strong human interface that bridges domain practice and technical solutions, generates reusable FAIR(agro)-enriched outputs (guidelines, tools, OERs)
*Limitations:* high dependence on data steward capacity and expertise, labour-intensive mediation effort that may not scale with growing service portfolio, insights reflect support-seeking users and are not fully representative of the broader community

**Interplay and Impact:** Translation means creating spaces where agricultural scientists and RDM experts can meet, exchange, and co-develop solutions. As these groups often "speak different languages," active mediation is needed to translate identified community needs into actionable RDM requirements. This involves evaluating and weighting needs, negotiating trade-offs between harmonisation (essential for interoperability) and flexibility (necessary for disciplinary diversity), and aligning FAIRagro's strategic focus accordingly. Equally important, maintaining this connection to the community is essential to produce FAIR resources and content that are both technically robust and practically relevant — forming the foundation for the next component, where FAIR(agro)-enriched solutions are communicated and FAIR(agro)-enriched support is provided.

## Component 3: Communicate FAIR(-agro) solutions and provide FAIR(-agro) enriched support

Communication is a critical enabler of cultural change. It ensures that the principles of FAIR RDM and the solutions developed within FAIRagro are not only disseminated but also understandable, relatable, and relevant to the main target groups and key stakeholders. FAIRagro follows a **multi-dimensional communication strategy**, combining different activities and formats to reach the main target groups and key stakeholders across disciplinary boundaries and institutional levels.

**Communication formats and media:** The FAIRagro Portal serves as the central information hub and one-stop entry point to all FAIRagro services and resources to disseminate tailored content to the three key stakeholder groups: Learners & Users, Multipliers & Decision Makers, and Developers & RDM Experts. Regular updates report on consortium developments, services, tools, and events. Complementing this, a monthly newsletter launched in late 2023 has steadily grown to nearly 300 subscribers by mid-2024, consolidating news and serving as a reliable channel to maintain community awareness. Strategic partnerships with professional societies and disciplinary associations (e.g. German Agricultural Research Alliance (DAFA), Society for Crop Science (GPW), and the German Soil Science Society (DBG)) play a pivotal role, as these organisations act as trusted multipliers with well-established communication channels. External communication is further amplified through blog posts, contributions to institutional newsletters, and participation in NFDI-wide campaigns such as *One NFDI*. Social media presence on Mastodon, LinkedIn, and BlueSky supports steady organic growth and community interaction, with content framed through storytelling and target group–specific narratives. A successful format is the widely read **FAIRagro legal blog** (https://fairagro.net/blog/), which translates complex RDM-related legal topics into accessible case stories.

*Strengths:* broad reach across target groups, increased visibility through society partnerships, growing newsletter and social media engagement, accessible formats and content for complex topics
*Limitations:* dependent on networks and subscriber engagement, high effort to maintain content quality, uneven impact across stakeholder groups, awareness does not guarantee adoption

**Events and in-person interaction:** Beyond digital formats, FAIRagro invests heavily in personal interaction to engage directly with its community. Activities range from lectures and workshops in institutional settings to field days, booths, and presentations at scientific conferences. The guiding

principle is to meet the community in their established environments — by participating in discipline-specific conferences and trusted events within research institutions, FAIRagro ensures visibility and relevance. Over the past two years, more than 50 such events have been conducted, directly reaching the community. The established FAIRagro event calendar provides structured information on upcoming events and includes links to event presentations and other published resources. Building on these experiences, a roadshow concept was developed and implemented to bring FAIRagro services directly to relevant institutions and target groups through hands-on sessions and practical demonstrations of FAIR(-agro) infrastructures and services.

*Strengths:* high visibility through presence in established disciplinary environments, strong trust-building via personal interaction, effective engagement through hands-on demonstrations
*Limitations:* resource-intensive and dependent on staff availability, limited scalability compared to digital formats, impact varies with event type and audience composition

**Helpdesk and networks:** The Helpdesk serves both as a communication channel and as a trust-building instrument, creating meaningful and reliable connections with the community. Many researchers first encounter FAIRagro through events or training and later turn to the Helpdesk for individual support. This continuity reinforces visibility, trust, and engagement. Personal interactions at conferences, workshops and training have been key in reaching over 40 institutions outside the FAIRagro universe on diverse topics of interest (Fig. 4). Importantly, the Helpdesk outputs (e.g. Repository List, Code Roadmap, Knowledge Base) are actively disseminated via communication formats and training activities, multiplying their reach. The Helpdesk is also embedded in the **Geo-Chem-Life Science Helpdesk Cluster**, a network of 10 NFDI helpdesks that exchange expertise on interdisciplinary questions. In addition, the **European Associated Data Steward Network Agriculture** (Schmidt et al., 2025; Fig. 7), co-coordinated by FAIRagro, establishes a national, low-barrier network of local Data Stewards linked to European counterparts, enabling structured cross-border exchange and long-term collaboration. Within the network, national contact points forward requests from or towards their associated data stewards, therefore connecting all participating institutions and broadening the pool of potential experts available to researchers seeking RDM support.

**<Figure 7>**

*Strengths:* builds trust through personal interaction, provides a trustworthy channel to promote FAIR(agro) services and best practices, benefits from expertise exchange within NFDI helpdesk clusters and the European Data Steward Network.
*Limitations:* network-based support depends on active participation and coordination across institutions, personal-contact pathways may bias reach toward already connected institutions, cross-network processes can increase complexity and coordination effort.

**Training and education:** To implement FAIR practices requires systematic capacity building. Therefore, FAIRagro invested in developing a comprehensive training concept (Boße & Lindstädt, 2025). Since the beginning of the funding phase, FAIRagro has conducted 32 training events, reaching more than 800 participants (as of November 2025) across institutions and target groups — from students to professors and department heads, and from university researchers to commercial plant breeders. Formats include introductory lectures, in-depth workshops, and hands-on sessions at scientific conferences — bringing RDM expertise directly to researchers who might not otherwise engage with the topic. Collaborations with agricultural universities enable tailored training for PhD students and early-career researchers, while joint activities with partners such as de.NBI (the German Network for Bioinformatics Infrastructure), the NFDI consortia for earth science, biodiversity and plant research (NFDI4Earth, NFDI4Biodiversity, NFDI4Plants) and PLANT2030 (the association of research activities in the field of applied plant research funded by the Federal Ministry of Education and Research (BMBF)), broaden the disciplinary scope. Content typically combines foundational modules (e.g. metadata documentation, repository publication, data management plans) with demand-driven workshops on specific topics. Increasingly, training also addresses legal challenges — such as data protection, copyright, and access and benefit sharing — delivered in collaboration with

other NFDI consortia. A discipline-specific focus ensures practical applicability. To this end, we have compiled and described a growing collection of freely accessible learning and teaching materials focused on RDM in agriculture and related disciplines (Boße et al., 2024). In addition, we develop our own Open Educational Resources to support both self-study and integration into institutional curricula. Correspondingly, the most pressing community needs are addressed through targeted training materials — for example, workshop resources on legal topics, developed in response to urgent demands in this area (Singson & Boße, 2025).

*Strengths:* broad reach across institutions and career stages, effective capacity building through diverse and discipline-specific formats, strong integration into established academic environments, trustworthy channel to promote FAIR(agro) practices, strengthened through collaborations with universities and NFDI consortia
*Limitations:* resource-intensive and dependent on trainer availability, limited scalability, varying engagement and reach across target groups, training alone cannot ensure sustained behavioural change

**Interplay and impact:** Making FAIR meaningful depends on identifying community needs and translating them into concrete outcomes, as described in the previous Cycle components. Building on these measures demonstrates that FAIRagro's communication goes far beyond one-way dissemination. Instead, it focuses on engaging with communities in their established environments, building trustworthy and meaningful connections, and embedding FAIR into disciplinary contexts and institutional spaces while showcasing tangible benefits. To further reach the growing community and implement FAIR practices effectively, the fourth component addresses the need to ensure that FAIR(agro)-enriched sound and outputs are not only produced but also delivered to and taken up by the community to anchor and sustain FAIR practices and drive cultural change.

## Component 4: Anchor and Sustain FAIR data practices

Anchoring and sustaining FAIR data practices requires moving beyond short-term projects and isolated measures toward embedded structures, shared responsibility, and institutionalised empowerment. The goal is to **institutionalize** FAIR practices, ensuring that they are not temporary add-ons but firmly anchored within organisations. This also means to **embed** FAIR approaches into disciplinary routines and institutional frameworks, where they become part of everyday research practice. Ultimately, the objective is to **sustain** these efforts through institutional uptake and long-term ownership. In this way, messages created through communication truly reach their intended audiences, building meaningful connections and transforming awareness into lasting commitment. FAIRagro addresses this challenge by combining governance mechanisms, institutional networks, and dedicated instruments that translate awareness into commitment and sustained engagement.

**Anchoring FAIRness through shared values.** The FAIRagro FAIRness and Openness Commitment (FAIRagro Consortium, 2025) provides a low-barrier entry point for individuals, projects, and institutions to declare their support for FAIR and Open Science principles. Adapted from NFDI4Earth (NFDI4Earth Consortium, 2024), the Commitment fosters visibility, transparency, and a shared understanding of data culture. It is designed to spark discussions within institutions, raise awareness in research groups, and promote a collective vision of openness as a norm rather than an aspiration—moving from **shared values to meaningful ownership**.

*Strengths:* low-barrier entry point, raises visibility of FAIR values, encourages institutional reflection, inspires change
*Limitations:* voluntary uptake limits coverage, awareness not equal to implementation

**Sustaining change through trusted agents.** The **FAIRagro Ambassador Network** embeds cultural change directly within institutions. Ambassadors — FAIRagro staff and principal investigators from partner organisations — act as visible and trusted contact points. As institutional insiders, they bridge between FAIRagro and their local contexts, tailoring and broadcasting FAIRagro content, and

channeling feedback back into the consortium. In doing so, they foster proximity, institutional ownership, and lasting engagement.

*Strengths:* trusted institutional contact points, strengthens local ownership, effective bidirectional communication
*Limitations:* engagement depends on individual capacity, uneven distribution across institutions

**Broadening participation through flexible partnerships.** The **Associated Partner status** enables external institutions, initiatives, and projects to join FAIRagro's network without the burden of full project membership. This lightweight format supports collaboration in training, standardisation, and consultation activities while anchoring broader participation and shared responsibility across the agricultural science landscape. It extends FAIRagro's reach to actors who might otherwise remain outside the consortium, thus strengthening sustainability through inclusiveness.

*Strengths:* flexible collaboration format, broadens participation beyond core consortium, strengthens inclusiveness
*Limitations:* limited binding obligations, variable depth of engagement

**From pilots to practices. Use Cases** describe RDM demands of the community and culminate in joint corporations between FAIRagro and agrosystem data or infrastructure users/providers. They serve as collaborative testbeds that not only generate practical, FAIR-compliant solutions but also disseminate them within their communities. By embedding FAIR practices into real research projects and disciplinary contexts, and by showcasing their tangible benefits, Use Cases increase visibility, foster trust, and accelerate the long-term transition toward collaborative and sustainable data management. Making these success stories visible — and keeping them close to the agricultural research community — has proven essential for broader engagement.

*Strengths:* embed FAIR in real research workflows, generate discipline-specific solutions, increase visibility through success stories
*Limitations:* resource-intensive, variable sustainability after project end

**Networks for sustainability.** Anchoring is further reinforced through **Helpdesk integration** and networking activities. The FAIRagro Helpdesk is embedded in the **Geo-Chem-Life Helpdesk Cluster** and co-coordinates the **Associated Data Steward Network Agriculture**. These networks ensure structured cross-border exchange, alignment of solutions, and long-term cooperation within national and European contexts.

*Strengths:* enable cross-institutional exchange, providing different expertise through networked and shared resources
*Limitations:* require continuous coordination, dependent on network activity and steward capacity

**Inclusiveness and strategic expansion.** Anchoring FAIR also means deliberately broadening FAIRagro's scope. This includes reaching underrepresented stakeholders and engaging strategically relevant neighbouring domains such as forestry, livestock and animal sciences, and food systems. By extending into these areas, FAIRagro fosters cross-disciplinary integration and ensures responsiveness to the evolving landscape of agricultural research.

**Interplay and Impact:** As Carole Goble aptly stated, "FAIR is a team sport." Successful team sports require clear goals, shared incentives, and visible rewards. In the context of FAIR, "winning" is not individual benefit but collective gain: better data quality, higher reusability, and more robust scientific outcomes. Anchoring FAIR practices therefore also depends on creating structures where collaboration is recognised and where contributing to shared infrastructure and Open Science is valued and rewarded. To achieve this, FAIRagro relies on openness, inclusiveness, and collaboration across the agricultural science ecosystem. The instruments of this component—Commitment, Ambassadors, Associated Partners, Use Cases, and Helpdesk networks—not only sustain practices

but also contribute across the entire Cycle. They build on **Component 1 (Identify and assess community needs)** by bringing in new communities and perspectives, thereby expanding and refining the understanding of needs. They reinforce **Component 2 (Translate)** by feeding back requirements and experiences into technical developments, and they expand **Component 3 (Communicate)** by acting as trusted multipliers and connectors. By closing the loop, FAIRagro both disseminates practices and integrates evolving needs from its growing community—transforming pilots into practices, and practices into sustained norms, thereby laying the foundation for long-term cultural change.

## From Metrics to Sustainability: Evaluating Long-Term Impact

Early evidence demonstrates that FAIRagro's measures are beginning to translate into observable achievements. Many of the DFG key performance indicators (KPIs) aim to capture this transition — ranging from straightforward metrics such as the number of events, trainings, and cooperation, to more complex indicators such as community engagement activities. In addition, the number of developed services and registered users provides insights into the practical implementation of FAIR practices (Amelung et al., 2023). Currently, all KPI are collected annually and as part of the interim and final reports. First results show steadily growing numbers, providing evidence that FAIRagro's efforts are becoming tangible and visible. Yet while quantitative indicators are useful, they only offer a partial view. To fully capture cultural change, it is equally important to tell success stories that illustrate the broader effects of individual measures — impacts that cannot be measured by numbers alone. One prominent example is the FAIRagro Roadshow in Bavaria at the Bayerische Landesanstalt für Landwirtschaft (LfL): it combined an initial needs assessment with a two-day hands-on training on RDM, including a live demonstration of services such as RDMO. In the aftermath, tailored DMP templates were developed for the institution, which have since been formally adopted and rolled out across the whole organisation — streamlining RDM processes and embedding FAIR practices into everyday routines. This example underlines the importance of systematically identifying needs, the sensitivity of translation work, and the necessity of tailored strategies to sustain FAIR practices over time. While KPIs capture visible outputs, the long-term sustainability of FAIRagro's cultural change efforts depends on how effectively different measures balance cost, effort and community impact. Across the Cycle, FAIRagro uses a diverse set of instruments — each with distinct investment profiles and benefits — whose combined effects ensure both breadth and depth of engagement.

**Community Surveys:** Surveys are comparatively low-cost and scalable, offering broad situational awareness with minimal staff time. Their main benefit lies in preventing misaligned development by revealing trends and priority needs early; however, they require regular updates to remain representative in fast-evolving domains.

**Use Cases**: Use Cases are resource-intensive measures that require sustained facilitation, domain expertise, and coordinated effort. Their return on investment is largely long-term and structural: co-created workflows, metadata mappings, and publication formats often persist beyond the project. Their focused scope enables deep, context-aware solutions, yet this specificity also entails the risk that outputs remain discipline-bound and offer only limited scalability across domains.

**Helpdesk:** Helpdesk consultations are labour-intensive but offer high value by building strong personal connections, serving as a trusted human interface to the community, and acting as a channel to promote FAIR solutions. Their cost-effectiveness increases as consultation materials, guidelines and how-tos are reused, allowing data stewards to focus on more complex, high-impact cases. A potential risk is that basic queries will increasingly be addressed by general AI tools, reducing the volume of entry-level RDM support requests handled through the Helpdesk.

**Training and Education:** Training is relatively labour-intensive and requires substantial expertise, yet its impact can be both sustainable and scalable. Trained participants often disseminate knowledge within their teams and institutions, thereby increasing the adoption of FAIR practices and the implementation of relevant services. Educational materials can be reused across settings and provide learning and teaching resources that extend well beyond individual training events. Sustainability is

further enhanced when training follows train-the-trainer approaches or when RDM topics become embedded in university curricula.

**Knowledge Base and Reusable Outputs**: Curating FAIRagro-enriched resources — such as guidelines, decision trees, repository recommendations, and workflows — requires upfront effort but generates very high long-term returns by supporting self-service learning and reducing repetitive support tasks. The cost-benefit improves further as materials are reused by partner institutions and integrated into curricula and or institutional RDM service portfolios.

**Events, Workshops and Roadshows**: In-person engagement demands significant organisational and staff resources, but it remains one of the most effective ways to build trust and visibly demonstrate the practical benefits of FAIR practices. The cost-benefit ratio improves when event materials and demonstrations are reused, adapted, and integrated into institutional structures.

**Strategic Communication (Portal, Newsletter, Blog, Social Media)**: Digital communication is relatively low-cost and provides continuous outreach, sustaining awareness across a large and heterogeneous community. While conversion rates vary, these channels are essential for amplifying FAIR(agros) content and outputs, guiding users to services and resources, and reinforcing recurring touchpoints at minimal marginal cost.

**Ambassador Network:** The Ambassador model requires ongoing coordination and relationship management but provides high-impact institutional anchoring with relatively low central investment. Its main benefit is multiplying FAIRagro's presence inside institutions, converting central messages into locally relevant practice and creating stable, long-lasting communication channels.

**Associated Partnership:** This low-barrier collaboration format demands little administrative overhead and offers a cost-effective way to broaden participation. While engagement depth varies, it fosters institutional commitment and provides access to new communities without requiring full consortium membership or funding..

# Discussion - Lessons learned and remaining challenges from fostering Cultural Change

## Lessons Learned from Fostering Cultural Change

### *1. Infrastructure is important but never sufficient*

FAIR research data management (RDM) in agrosystem sciences cannot be achieved through technical solutions alone. Infrastructures, standards and services form the essential backbone for making data findable, accessible, interoperable and reusable (FAIR) (Wilkinson et al., 2016), yet their success ultimately depends on cultural acceptance, usability and alignment with real research practices. FAIRagro's early phase illustrates this clearly: despite notable progress in infrastructure and service development, many services remained only partially embedded in daily scientific workflows, and their uptake was modest. Evaluations by the Community Advisory Board (CAB) confirmed that technical progress must be complemented by continuous engagement, user-centered design, and context-specific guidance. This reflects Nosek's (2019) view that infrastructure makes change possible, but does not make it happen.

The Community Engagement and Empowerment Cycle responds to this challenge by placing equal emphasis on infrastructure and community processes. It ensures that needs are actively captured, contextualised, and transformed into actionable requirements; that solutions are made tangible and relevant; and that support structures are embedded where researchers actually work. FAIRagro's experience aligns with broader Open Science insights: without co-creation, interface usability, and authentic engagement, technical developments alone rarely lead to behavioural change.

### *2. Cultural change is a socio-technical challenge; isolated interventions are insufficient*

RDM cultural change is multidimensional and cannot be achieved through single interventions. FAIRagro implemented a broad range of instruments — Use Cases, Helpdesk support, training, dissemination activities, surveys and stakeholder engagement — yet their impact emerges only through integration, not in isolation. Individual instruments shed light on specific challenges, but when insights from Use Case applications, Helpdesk tickets, surveys and event-based feedback were synthesised, they collectively revealed a more complete and actionable picture of community needs. A similar pattern becomes visible in communication activities. Information alone rarely really reaches or activates the intended audiences; communication becomes effective only when it is systematically connected to mechanisms that ensure that messages are targeted, contextualised and supported by practical follow-up measures. Cultural change therefore requires interventions that are conceived together and mutually reinforcing rather than parallel or fragmented efforts.

This pattern is not unique to FAIRagro; it is characteristic of the broader RDM landscape. Across many initiatives and projects, efforts often address only one piece of the FAIR puzzle, and their full potential unfolds only when they are aligned within a coherent strategy. Initiatives like FAIRagro are therefore particularly important, as they help to join forces and foster collaboration across national RDM initiatives, project-based developments, and institutional activities. Data Stewards, in particular, play a key role as connectors: they build bridges between infrastructure development and domain science, translate requirements in both directions, and help weave together otherwise fragmented efforts. Similarly, ambassadors and other local intermediaries contribute by translating, contextualising, and promoting FAIR practices within institutional settings. Together, these roles are essential for integrating diverse RDM efforts and demonstrate that collaboration — rather than isolated interventions — is the true driver of progress toward FAIR RDM.

### *3. Effectiveness of change depends on strong connections and translation*

While institutional conditions shape cultural change at the national level, long-term sustainability also depends on the ability of agricultural RDM to interface with international standards, infrastructures and legal frameworks. Effective cultural change requires actors who can navigate between the worlds of domain science, research data management, and technical infrastructure. Agricultural researchers, RDM specialists, and infrastructure developers often operate with different priorities, vocabularies and expectations, creating substantial "translation gaps" between these groups (Senft et al., 2022). The step from a researcher's informal description of data handling related challenges to a structured requirement or actionable Git issue illustrates this gap. As Borgman (2015) emphasises, the organisational, interpretative and interpersonal work required for effective RDM is frequently underestimated — even though it is essential to connect technical developments with real-world scientific practice.

The Community Engagement and Empowerment Cycle addresses this challenge by explicitly recognising "translation" as a critical and often underestimated component of FAIR implementation. It integrates targeted measures and activities that operationalise this translation work in practice. Use Case working groups create structured spaces in which agricultural researchers and technical experts jointly develop solutions — from metadata harmonisation to workflow integration — thereby transforming domain-specific needs into actionable technical requirements. Similarly, the Helpdesk channels individual community requests into service and infrastructure development and, in turn, translates resulting technical outputs back into community-appropriate language, producing FAIR-enriched resources that support uptake and implementation.

Such translation work is frequently undervalued in RDM initiatives, even though it is essential in both directions: from users to service and infrastructure developers, and from technical solutions back into applicable practice (OECD, 2020). This assessment is echoed in the Data- und Digital-Science-Community (DaDiSC) Positionspapier (2025), which emphasises that infrastructures become effective only when the community is enabled to use them, that feedback, methodological competence and co-design are critical, and that persistent "Scharnierstrukturen" are needed to connect disciplinary practices with service and infrastructure development. Translation is not only a technical task but also a cultural one. Effective FAIR solutions must be contextualised for different user groups, embedded in their workflows and grounded in the day-to-day realities of scientific work, and communicated through diverse and accessible formats — from technical documentation and reproducible workflows to guidelines, checklists, training materials and open educational resources. This aligns with insights from the broader Open Science literature, which emphasises that transparency, reproducibility and openness require deep integration into researchers' daily routines, not merely the availability of suitable infrastructure (Nosek et al., 2015; Munafò et al., 2017). Only when services become tangible and embedded into the "researcher's journey," can they support sustainable adoption and the normalisation of FAIR practices.

### *4. Shared values and trust as socio-cultural prerequisites for change*

Trust, shared values and a common mission form the socio-cultural foundation on which any sustainable change in research data management must rest. Social Capital Theory (Bourdieu, 1986; Coleman, 1988; Putnam, 1993) highlights that collective action depends on shared norms, mutual expectations and relationships of trust that enable actors to coordinate behaviour. In fragmented research domains such as the agrosystem sciences, where disciplinary traditions and institutional contexts vary widely, establishing such shared understandings is essential for enabling collaboration across boundaries. FAIRagro's bottom-up, domain-centred approach directly addresses these prerequisites. By rooting its activities in the scientific cultures, practices and epistemic traditions of the agrosystem sciences, it created the trust, relevance and proximity needed for FAIR practices to be cultivated and grow. Instruments such as the FAIRness & Openness Commitment, the Ambassador Network and the Associated Partner programme cultivate shared values, strengthen credibility and

create recognisable points of contact that help build trust within and across institutions. CAB evaluations underline that researchers and institutions are more willing to adopt FAIR practices when they perceive FAIRagro as aligned with their disciplinary identity and values, rather than as an external compliance mechanism. Without common norms and trust-based relationships, technical infrastructures and policy incentives are unlikely to translate into changed behaviour. Shared values are therefore not the outcome but the precondition for meaningful engagement and subsequent shifts in practice.

A further dimension of trust concerns the perceived long-term reliability of infrastructures and services. Cultural change requires substantial investment of time, adaptation and learning; such investments occur only when researchers believe that infrastructures will persist, be maintained and evolve in a predictable way. The 2025 structural evaluation of the NFDI by the German Science Council explicitly highlights that trust in transformation processes depends on stable governance, sustainable operating models and coherent long-term strategies across consortia (Wissenschaftsrat, 2025). These concerns are echoed by the NFDI consortia themselves, who underline the need for coordinated base services, sustainable operating models and clear long-term strategies (Konsortialversammlung NFDI, 2024). At the same time, trust-building has been supported by the NFDI and similarly FAIRagro's domain-centred strategy, which anchors infrastructures and services within familiar disciplinary contexts. Proximity to established actors, recognised institutions and existing scientific communities has created credibility and strengthened relational trust within the agrosystem sciences. The forthcoming transformation and consolidation processes within the NFDI therefore face a delicate balance: while streamlining, coordination and shared base components are needed to increase efficiency and sustainability, it is equally essential to preserve and further strengthen the domain-specific trust-building elements that enabled broad community uptake in the first place. Consolidation should not come at the expense of proximity, disciplinary relevance or the relationships of trust that underpin cultural change.

### *5. Sustainable change requires shared ownership and active responsibility*

A second theoretical anchor is the Community Engagement Continuum (CDC, 2011), which conceptualises engagement as an evolving process from *inform* → *consult* → *involve* → *collaborate* → *empower*. FAIRagro has put this continuum explicitly into practice: its Engagement and Empowerment Cycle reflects that communication alone is not sufficient; meaningful progress requires co-creation with the community. Empowerment is the ultimate goal, because RDM cannot be implemented *for* the community, but only together *with* it. Sustainable cultural change requires that actors at all levels—from individuals to institutions and global stakeholders—assume ownership and active responsibility. Finally, the Cycle provides a holistic perspective by integrating different FAIRspectives: FAIR for me at the level of the individual researcher (placing the researcher's journey at the centre), FAIR for us at the institutional level, and FAIR for all at the systemic and ultimately global level. Only by linking these perspectives can cultural change be consistently pursued across individual, institutional, and structural dimensions (Goble, 2024). This logic aligns with Goble's observation that "FAIR is a team sport": meaningful progress emerges only when infrastructure and service providers deliver tailored solutions, researchers actively engage and adopt FAIR practices, and institutions create the conditions and take responsibilities that enable these behaviours to take root.

## The Way Ahead: Structural Challenges and Conditions for Sustainable FAIR adoption

### *Cultural change: Moving from Metrics to Norms and Institutional Practice*

Despite substantial progress, long-term cultural change towards FAIR RDM is challenged by persistent structural inequalities across the agricultural research landscape. Smaller institutions, applied research organisations, or individual researchers without dedicated RDM support remain difficult to reach and risk being marginalised in large-scale infrastructure initiatives (Chamorro-Padial et al., 2024). Findings from the FAIRagro Cycle confirm this: the Identify and Translate phases showed that needs are unevenly distributed, and that active participation remains concentrated among well-resourced institutions and established networks.

Sustaining engagement over time is an additional challenge. Initial enthusiasm may diminish if researchers do not experience tangible benefits or if institutional support structures remain fragile. Embedding FAIR practices into institutional routines — not only into project-based activities — is therefore essential for enabling community participation to mature into stable behavioural norms. These challenges emphasise the need to strengthen the *Anchor* component of the Cycle, ensuring that engagement is not episodic but reinforced through institutional structures, training and continued support.

The 2025 structural evaluation of the National Research Data Infrastructure (NFDI) by the German Science Council (Wissenschaftsrat, 2025) highlights governance, sustainability and efficiency as central criteria for the long-term viability of research data infrastructures. The evaluation underscores that cultural change cannot be sustained if the technical backbone is fragmented, insufficiently coordinated or economically inefficient. Long-term sustainability requires shared, high-quality and widely acknowledged infrastructures and services that provide a stable foundation for domain-specific engagement. The FAIRagro Cycle demonstrates how trust, proximity and disciplinary relevance can foster engagement, but these trust-building elements depend on the perceived long-term reliability of infrastructures and operators. As the NFDI enters its consolidation and transformation phase, a key challenge will be balancing efficiency gains through shared base services with the preservation of domain-specific structures that enable proximity, credibility and contextual relevance. Consolidation must therefore strengthen — rather than jeopardise — the socio-cultural conditions that made engagement possible in the first place.

Sustainable cultural change also depends on aligning bottom-up engagement with institutional incentives and broader regulatory frameworks. As Nosek (2019) argues, cultural change requires coordinated progression across multiple layers: infrastructure makes FAIR possible; interfaces make it easy; communities make it normative; incentives make it rewarding; and policies make it required. FAIRagro has progressed strongly in the first three layers, yet institutional incentives remain uneven. Many agricultural research institutes have RDM or data policies, but their enforcement, scope and linkage to concrete rewards for researchers vary significantly. This complexity is amplified in the German research system, where bureaucracy and over-regulation can impose barriers to adoption. Cultural change requires supportive policies that balance expectations with feasibility, avoiding administrative burdens that disproportionately affect smaller organisations or researchers without RDM support.

The broader scientific ecosystem illustrates the potential impact of stronger incentives and regulations. In scholarly publishing, for example, the evolution from optional data availability statements to mandatory data publication requirements has significantly shifted researcher behavior (Colavizza et al., 2020; Federer et al., 2018). Similar mechanisms could be strengthened within agricultural science through research policies, funder requirements, and institutional mandates. However, the debate remains controversial: “carrot and stick” approaches — combining incentives

and sanctions — are widely discussed in Open Science governance, with differing views on their effectiveness and acceptance (Fecher & Friesike, 2014).

For FAIRagro, this debate underscores the necessity of moving beyond purely voluntary measures and strengthening its role at the interface of community-driven engagement and institutional and governmental policy frameworks. Additional regulatory elements and incentive structures could help to manifest and reinforce cultural change, ensuring that FAIR practices are not only encouraged but also embedded as durable norms. Current political developments such as the forthcoming Research Data Act (BMBF, 2023; NFDI e.V., 2023 (Stellungnahme Forschungsdatengesetz)) and the federal government's GovData and Open Data strategy point in this direction. Both initiatives emphasize that publicly funded research data must be made openly available, a requirement that equally applies to the departmental research institutions represented within the FAIRagro consortium.

### *Beyond the Local: The Researcher's Journey in an Internationally Aligned FAIR Ecosystem*

Agricultural research intersects with several closely related domains — including environmental and Earth system sciences, ecology and biodiversity research, environmental and agricultural engineering, and socio-economic sciences — all of which generate overlapping data types and share similar challenges in semantics, interoperability and long-term data stewardship, creating interdependencies that cannot be addressed within isolated disciplinary or national silos. Without systematic coordination, these overlaps risk producing divergent standards, duplicated infrastructures and incompatible metadata practices, undermining long-term interoperability.

To address these challenges, harmonisation must extend across vocabularies, semantic frameworks and metadata efforts. Established international infrastructures and initiatives — such as the Research Data Alliance (RDA), ELIXIR, the European Open Science Cloud (EOSC), and domain-specific standards like AGROVOC and MIAPPE — provide essential venues for developing and aligning such frameworks. In line with the FAIR principles (Wilkinson et al., 2016), which emphasise community-agreed standards and interoperable infrastructures, the interplay of research data infrastructures at institutional, national and international levels is equally crucial. This alignment ensures the interoperability, visibility and scientific relevance of FAIRagro's developments — including services such as the FAIRagro Search Hub — within the global agricultural research environment. Addressing interoperability also requires consideration of interdisciplinary and cross-domain data sources, such as PANGAEA for environmental and biodiversity data, or global agricultural infrastructures like CGIAR's GARDIAN for instance, which provide essential context and linkage points for agrosystem research. As EOSC continues to evolve, interoperability will become even more critical, with EOSC expected to play an increasingly central role in connecting disciplinary data spaces and providing shared data management services. For FAIRagro, linking agricultural RDM with these broader ecosystems is fundamental to ensuring that solutions developed at the national or disciplinary level can scale, interoperate and remain sustainable in a global research environment. Cross-border collaboration is further supported by community-driven networks such as the European Associated Data Steward Network Agriculture, which facilitates exchange among data stewards, promotes shared practices in metadata, licensing and data handling, and helps harmonise implementation across institutional and national boundaries.

The legal environment adds an additional layer of complexity. The heterogeneous legal characterisation of research data continues to generate uncertainty, making it necessary to coordinate bottom-up domain expertise with evolving regulatory frameworks. The EU Data Governance Act (Regulation (EU) 2022/868) and the Data Act (Regulation (EU) 2023/2854) introduce

new models for data access, re-use and connected-device data — developments that directly affect agrosystem science fields reliant on sensor networks, precision agriculture and digitally integrated on-farm processes (European Union, 2022 and 2023). Ensuring that FAIRagro’s practices and infrastructures align with these European-level regulatory developments will be essential for enabling legally robust, internationally interoperable and future-proof FAIR implementations. Taken together, these factors illustrate that sustainable FAIR adoption requires not only domain-centred engagement and robust national structures, but also coherent alignment with international standards, infrastructures and regulatory developments.

## Conclusions

The evaluations of the FAIRagro Community Advisory Board and continuous community feedback revealed three central gaps — an engagement gap, an integration gap and a guidance gap — that motivated the development of the Community Engagement and Empowerment Cycle. The Cycle provides a structured response to these shortcomings by operationalising the prerequisites for sustainable cultural change: needs must be actionable, solutions must be tangible, and communication must be embedded in the environments where researchers work.

Through its four components — *Identify*, *Translate*, *Communicate* and *Anchor* — the Cycle connects technical development with socio-cultural processes and transforms fragmented interventions into a coherent strategy. The experience gained in FAIRagro highlights five key lessons:

1. Infrastructure is essential but never sufficient; adoption requires usability, contextualisation and co-creation.
2. Cultural change is a socio-technical challenge that cannot be achieved through isolated measures but only through coordinated, mutually reinforcing activities.
3. Effective change depends on translation and strong connectors who bridge domain science, RDM and infrastructure development .
4. Shared values and trust are socio-cultural prerequisites that enable collaboration across institutional and disciplinary boundaries.
5. Sustainable change requires shared ownership and active responsibility — FAIR is a team sport, that depends on the contributions of researchers, institutions and infrastructures alike.

Taken together, these insights demonstrate that cultural change must be pursued across all three levels of the research ecosystem: the individual level, where daily practices and skills evolve; the institutional level, where policies, incentives and support structures shape behavioural norms; and the systemic or global level, where interoperability, standards and international collaboration enable sustainable and scalable FAIR practices. By addressing all three dimensions simultaneously, the Cycle provides a replicable and scalable model for fostering cultural change in agrosystem research and beyond.

Its approach strengthens the link between user needs and technical implementation, supports the creation of trusted and durable RDM structures, and helps anchor FAIR practices across institutions, disciplines and national borders. Ultimately, the FAIRagro approach contributes to a culture of collaborative RDM and Open Science, enabling agricultural sciences to advance innovative research and deliver actionable solutions to global challenges such as food security, climate resilience and sustainability.

## References


Ali B, Dahlhaus P. The role of FAIR data towards sustainable agricultural performance: a systematic literature review. *Agriculture*. 2022;12(2):309. https://doi.org/10.3390/agriculture12020309

Amelung L, Anthofer V, Danabalan R, Demandt É, Ebert B, Elschner E, et al. White paper: interim report reference. *Zenodo*. 2023. https://doi.org/10.5281/zenodo.7688729

Arnstein SR. A ladder of citizen participation. J Am Inst Plann. 1969;35(4):216–24.

Bernard L, Brück M, Busse C, Engel J, Eufinger J, Ewert F, et al. Memorandum of understanding of NFDI consortia from Earth-, Chemical and Life Sciences to support a network called the Geo-Chem-Life Science Helpdesk Cluster (Version 1). *Zenodo*. 2025. https://doi.org/10.5281/zenodo.15065070

Beyer F, Vedder L, Singson LS, Sahwan W, Schmidt M. Publishing research code FAIR – a roadmap (1.0). *Zenodo*. 2025. https://doi.org/10.5281/zenodo.14772749

Boße S, Lindstädt B. FAIRagro concept for training, education and educational resources. Müncheberg: FAIRagro; 2025. Available from: https://doi.org/10.4126/FRL01-006511849

Boße, S, Rey Mazón, E, Sennhenn, A, Vedder, L, Schneider, G. Metadata in agricultural research practice. *Fachrepositorium Lebenswissenschaften*. 2025. Available from: https://doi.org/10.4126/FRL01-006490238

Boße S, Singson LS, Sahwan W, Rey-Mazón E, Vedder L. How to learn and teach research data management in agrosystem research – a FAIRagro collection (1.0). *Zenodo*. 2024. https://doi.org/10.5281/zenodo.11148701

Bundesministerium für Bildung und Forschung (BMBF). Eckpunkte für ein Forschungsdatengesetz. Berlin: BMBF; 2023 [cited 2025 Sep 3]. Available from: https://www.bmftr.bund.de/SharedDocs/Downloads/DE/gesetze/forschungsdatengesetz/sonstige/Eckpunktepapier.pdf

Centers for Disease Control and Prevention (CDC). Principles of community engagement. 2nd ed. Atlanta (GA): CDC/ATSDR Committee on Community Engagement; 2011. Available from: https://ictr.johnshopkins.edu/wp-content/uploads/2015/10/CTSAPrinciplesofCommunityEngagement.pdf

Chamorro-Padial J, García R, Gil R. A systematic review of open data in agriculture. Comput Electron Agric. 2024; 219:108775.

Colavizza G, Hrynaszkiewicz I, Staden I, Whitaker K, McGillivray B. The citation advantage of linking publications to research data. PLOS ONE. 2020;15(4):e0230416.

Coleman JS. Social capital in the creation of human capital. Am J Sociol. 1988;94:S95–120.

Data- und Digital-Science-Community (DaDiSC). Positionspapier zur Zukunft der Data- und Digital-Science- Community. Version 1.1. 2025. Available from: https://doi.org/10.5281/zenodo.17475528

European Commission. Strategic research and innovation agenda (SRIA) of the Agriculture of Data Partnership. Brussels: Directorate-General for Research and Innovation; 2023. Available from: https://research-and-innovation.ec.europa.eu/system/files/2023-08/AgData%20SRIA%20final_version.pdf

European Union. Regulation (EU) 2022/868 of the European Parliament and of the Council of 30 May 2022 on European data governance and amending Regulation (EU) 2018/1724 (Data Governance Act). Official Journal of the European Union. 2022; L152:1–44.

European Union. Regulation (EU) 2023/2854 of the European Parliament and of the Council of 13 December 2023 on harmonised rules on fair access to and use of data (Data Act). Official Journal of the European Union. 2023; L2023:1–131.

FAIRagro Consortium. FAIRagro Fairness and Openness Commitment. 2025. Zenodo. https://doi.org/10.5281/zenodo.14925202

Fecher B, Friesike S. Open science: one term, five schools of thought. In: Bartling S, Friesike S, editors. *Opening science*. Cham: Springer; 2014. p. 17–47.

Federer LM, Belter CW, Joubert DJ, Livinski A, Lu Y-L, Snyders LN, et al. Data sharing in PLOS ONE: an analysis of Data Availability Statements. *PLOS ONE*. 2018;13(5):e0194768.

Finger R, Henningsen A, Höhler J, Huber R, Rommel J, Grebitus C. Open science in agricultural economics. *Q Open*. 2024;5(3):qoae029. https://doi.org/10.1093/qopen/qoae029

Geels FW. Technological transitions as evolutionary reconfiguration processes: a multi-level perspective and a case-study. Res Policy. 2002;31(8–9):1257–74.

Goble C. Insights from a UK bioscience infrastructure [Internet]. Presented at: EOSC Symposium; 2024 Oct; Berlin, Germany. EOSC; 2024 [cited 2025 Sep 3]. Available from: https://eosc.eu/wp-content/uploads/2024/10/Goble_Insights-from-a-UK-Bioscience-Infrastructure.pdf

Iaia V. The Elephant in the Room of EU Copyright Originality: Time to Unpack and Harmonize the Essential Requirement of Copyright.' *The Journal of World Intellectual Property*, 28, 471–490. 2025. https://doi.org/10.1111/jwip.12343

Konsortialversammlung des Vereins Nationale Forschungsdateninfrastruktur (NFDI) e.V. Status quo und Zukunft der NFDI – eine Perspektive der Fachkonsortien. *Zenodo*. 2024. https://doi.org/10.5281/zenodo.14277471

Munafò MR, Nosek BA, Bishop DVM, Button KS, Chambers CD, du Sert NP, et al. A manifesto for reproducible science. *Nat Hum Behav*. 2017;1:0021.

NFDI e.V. Stellungnahme zum Forschungsdatengesetz [Internet]. 2023 May 10 [cited 2025 Sep 3]. Available from: https://www.nfdi.de/wp-content/uploads/2023/05/NFDI-Stellungnahme-zum-Forschungsdatengesetz.pdf

NFDI4Earth Consortium. NFDI4Earth Fairness and Openness Commitment (1.0). 2024. NFDI4Earth Community on Zenodo. https://doi.org/10.5281/zenodo.10123880

Nosek BA. Strategy for culture change [Internet]. Charlottesville (VA): Center for Open Science; 2019 Jun 11 [cited 2025 Sep 3]. Available from: https://www.cos.io/blog/strategy-for-culture-change

Nosek BA, Alter G, Banks GC, Borsboom D, Bowman SD, Breckler SJ, et al. Promoting an open research culture. *Science*. 2015; 348(6242):1422–5.

OECD. Building digital workforce capacity and skills for data-intensive science. Paris: OECD Publishing; 2020. (OECD Science, Technology and Industry Policy Papers, No. 90). doi:10.1787/e08aa3bb-en.

Putnam RD. *Making democracy work: civic traditions in modern Italy.* Princeton (NJ): Princeton University Press; 1993.

Rey-Mazón E, Vedder L, Beyer F, Schmidt M. Recommended repositories by the FAIRagro Helpdesk for agrosystem research (1.0). *Zenodo*. 2024. https://doi.org/10.5281/zenodo.11144471

Rosenzweig C, Jones JW, Hatfield JL, Ruane AC, Boote KJ, Thorburn P, et al. The Agricultural Model Intercomparison and Improvement Project (AgMIP): protocols and pilot studies. *Agric For Meteorol*. 2013; 170:166–82. https://doi.org/10.1016/j.agrformet.2012.09.011

Ryan M, Atik C, Rijswijk K, et al. The future of agricultural data-sharing policy in Europe: stakeholder insights on the EU Code of Conduct. *Humanit Soc Sci Commun*. 2024;11:1197. https://doi.org/10.1057/s41599-024-03710-1

Schmidt M, Albers J, van Heijnsbergen E, Ní Fhlaithearta S, Belin K, Lindroos H, et al. Associated data stewards as means to broaden domain-specific expertise – a European perspective for the agricultural sciences. In: IDCC 2025; 2025 May 12; Den Haag, Netherlands. *Zenodo*. 2025. https://doi.org/10.5281/zenodo.15386401

Senft M, Stahl U, Svoboda N. Research data management in agricultural sciences in Germany: we are not yet where we want to be. *PLOS ONE*. 2022;17(9):e0274677.

Sennhenn A, Hoffmann C, Srivastava AK, Heuermann MC, Neumann K, Fluck J, et al. Approved proposals of the 1st FAIRagro Use Case Call 2024. *Zenodo*. 2025. https://doi.org/10.5281/zenodo.14761793

Sennhenn A, Hoffmann C, Tapken H., Mühlhaus T, Werban U, Schütze C. Approved Proposals of the 2nd FAIRagro Use Case Call 2025. *Zenodo*. 2025. https://doi.org/10.5281/zenodo.17589618

Singson L, Boße S, Schlimbach A, Kuppler J, Tschink D. Datenschutz auf dem Silbertablett: Trainingsmaterial. Müncheberg: FAIRagro; 2025. Available from: https://doi.org/10.4126/FRL01-006402743

Specka X, Martini D, Weiland C, et al. FAIRagro: ein Konsortium in der Nationalen Forschungsdateninfrastruktur (NFDI) für Forschungsdaten in der Agrosystemforschung. *Informatik Spektrum*. 2023;46:24–35. https://doi.org/10.1007/s00287-022-01520-w

Uhlott J, Sennhenn A, Möller M. FAIRagro survey: data quality in the application of agricultural system data. *Zenodo*. 2024. https://doi.org/10.5281/zenodo.11612449

Wilkinson MD, Dumontier M, Aalbersberg IJ, Appleton G, Axton M, Baak A, et al. The FAIR Guiding Principles for scientific data management and stewardship. Sci Data. 2016; 3:160018. doi:10.1038/sdata.2016.18

Wissenschaftsrat. Strukturevaluation der Nationalen Forschungsdateninfrastruktur (NFDI). Berlin: Wissenschaftsrat; 2025. (Drs. 2627-25). Available from: https://www.wissenschaftsrat.de/

# Figures and Tables & Legends

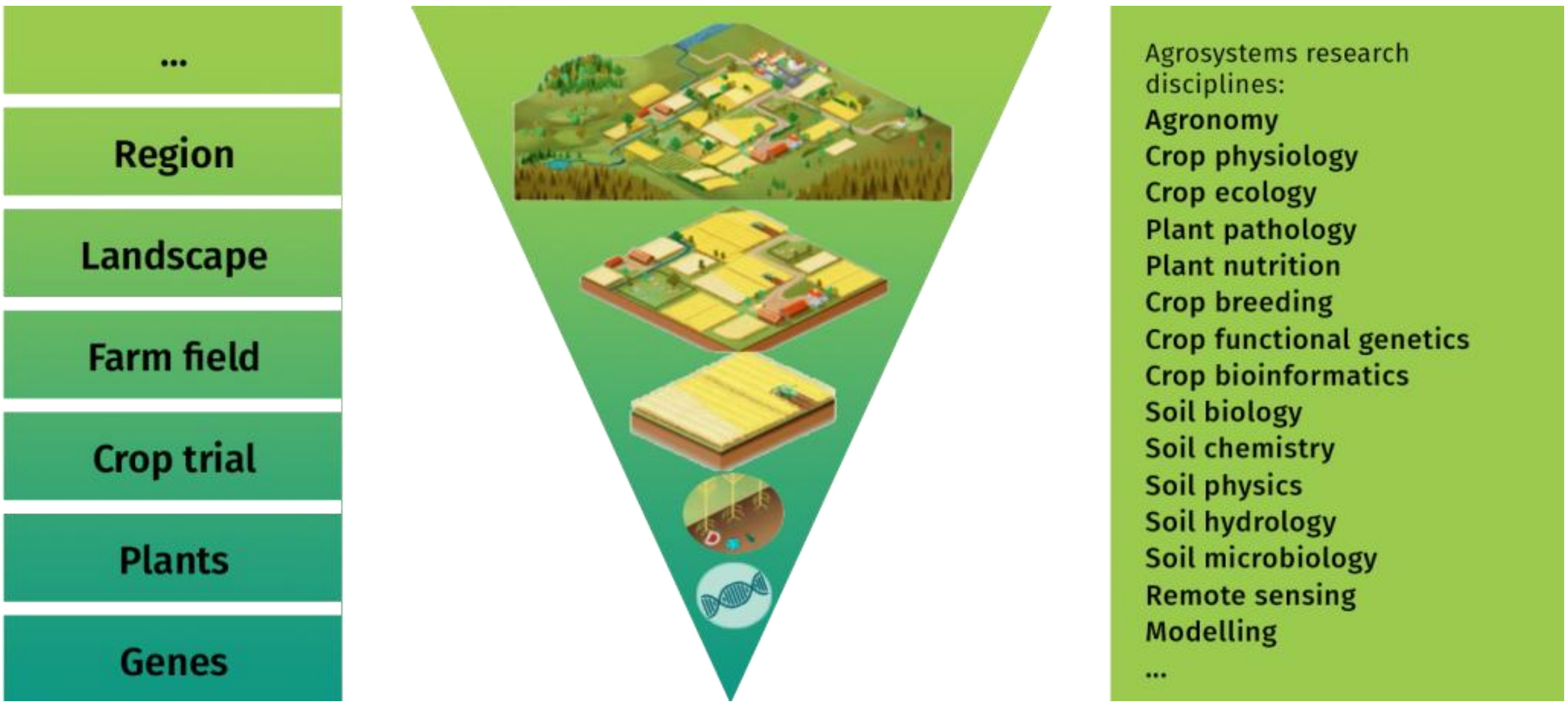


**Figure 1:** FAIRagro scales and disciplines

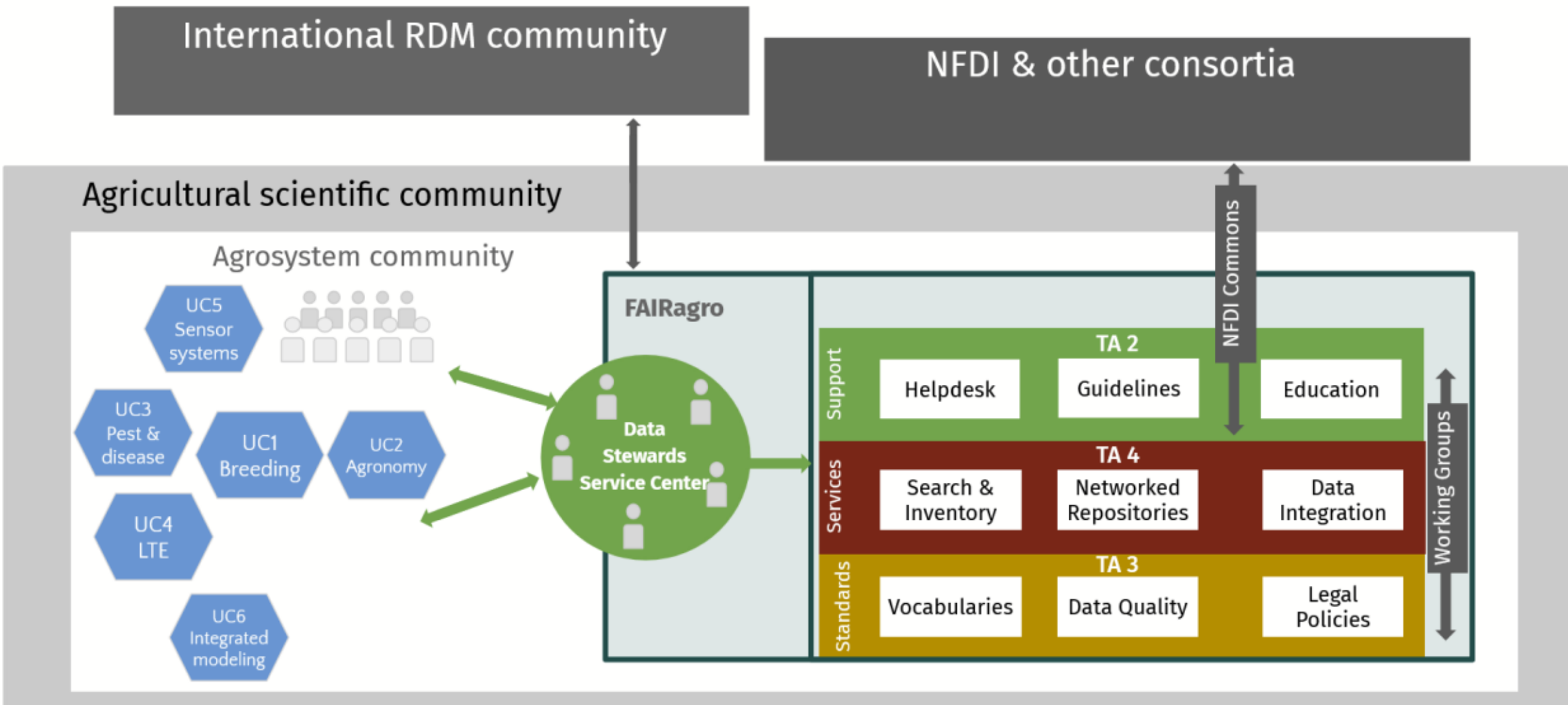


**Figure 2:** FAIRagro concept and structure

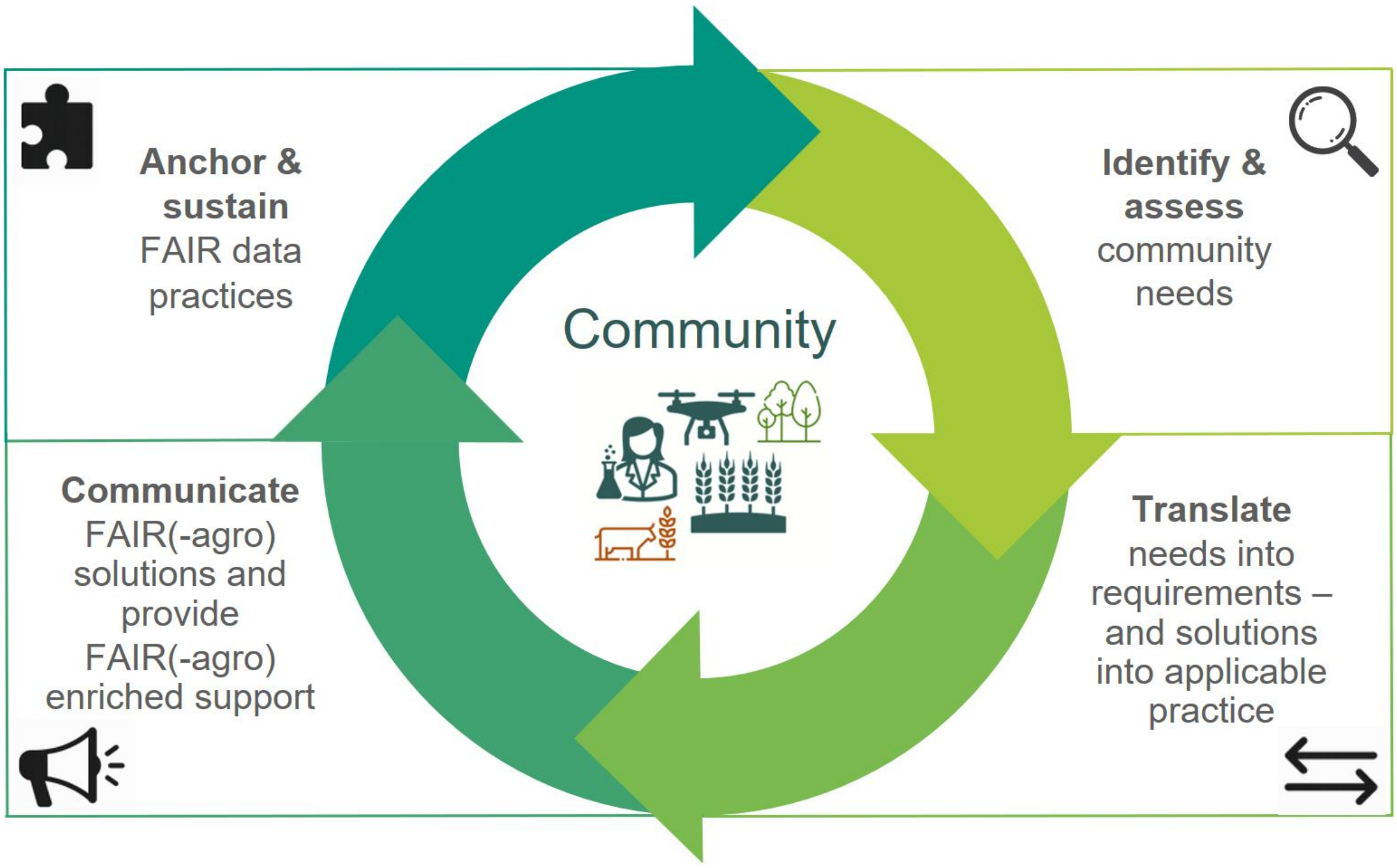


**Figure 3:** FAIRagro Community Engagement and Empowerment Cycle

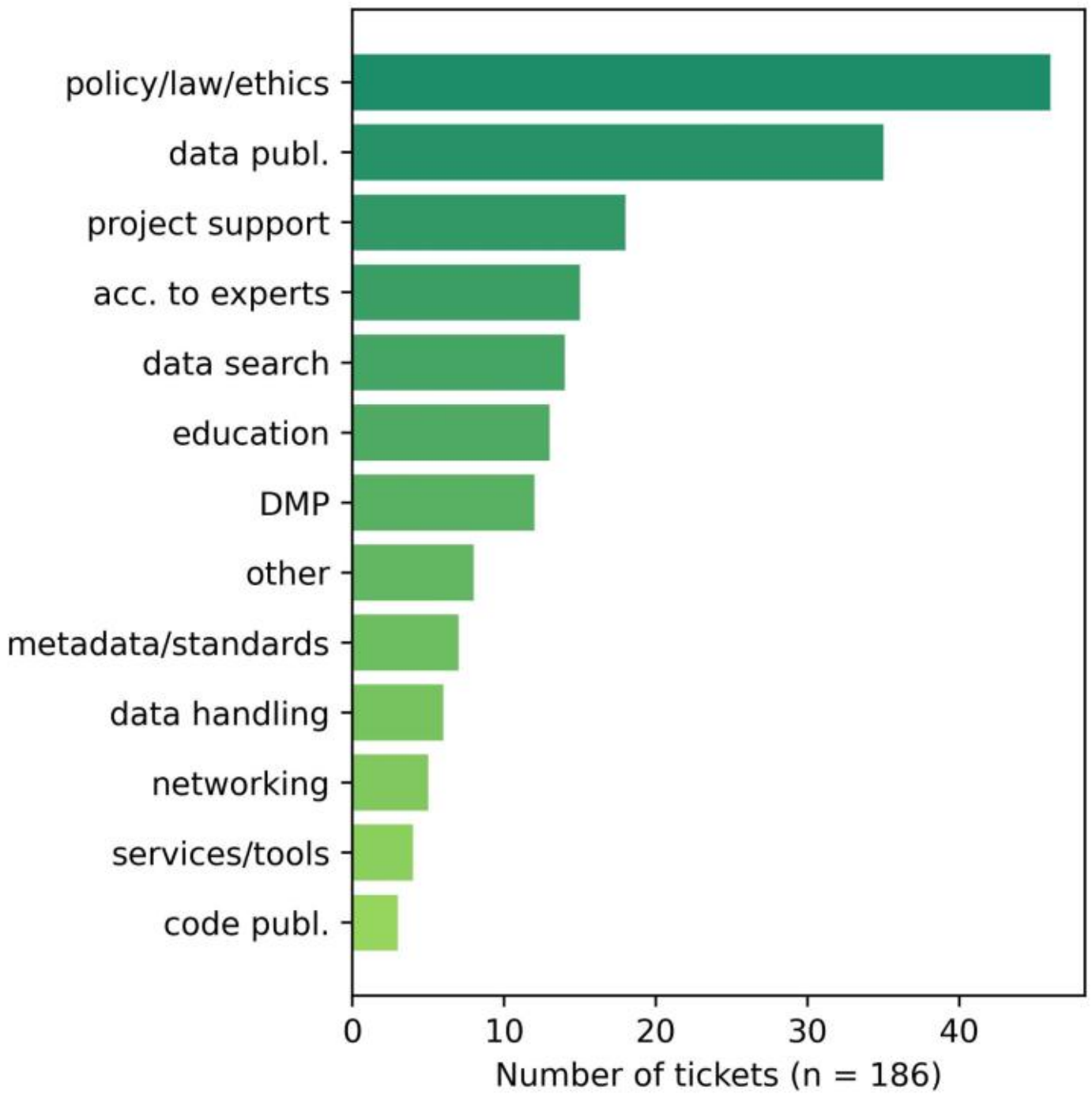


**Figure 4:** Request categories of the FAIRagro Helpdesk. Represented are tickets from March 2023 to September 2025. “DMP” stands for data management plans.

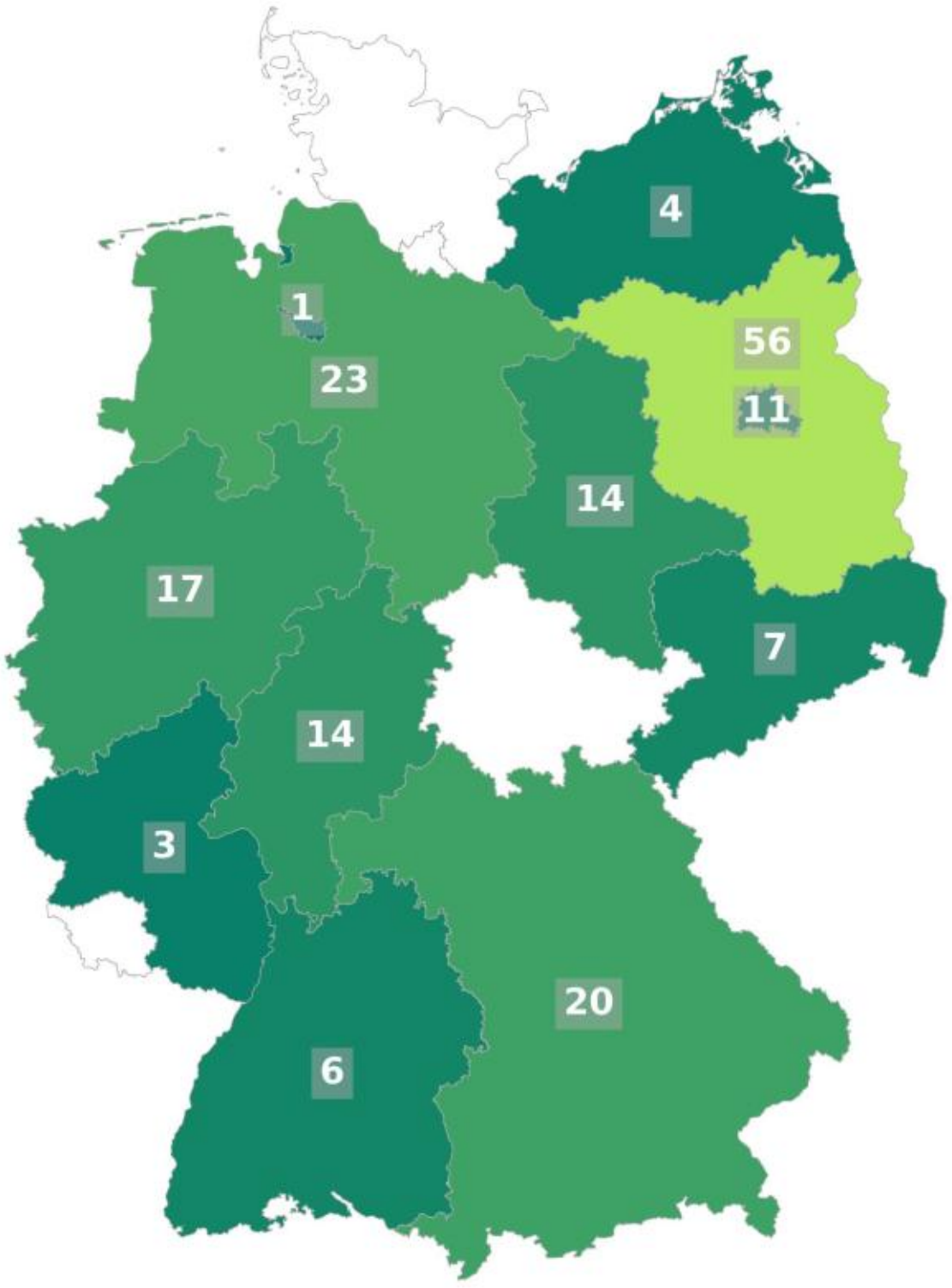


**Figure 5:** Number of tickets of the FAIRagro Helpdesk per federal state. Represented are tickets from March 2023 to September 2025.

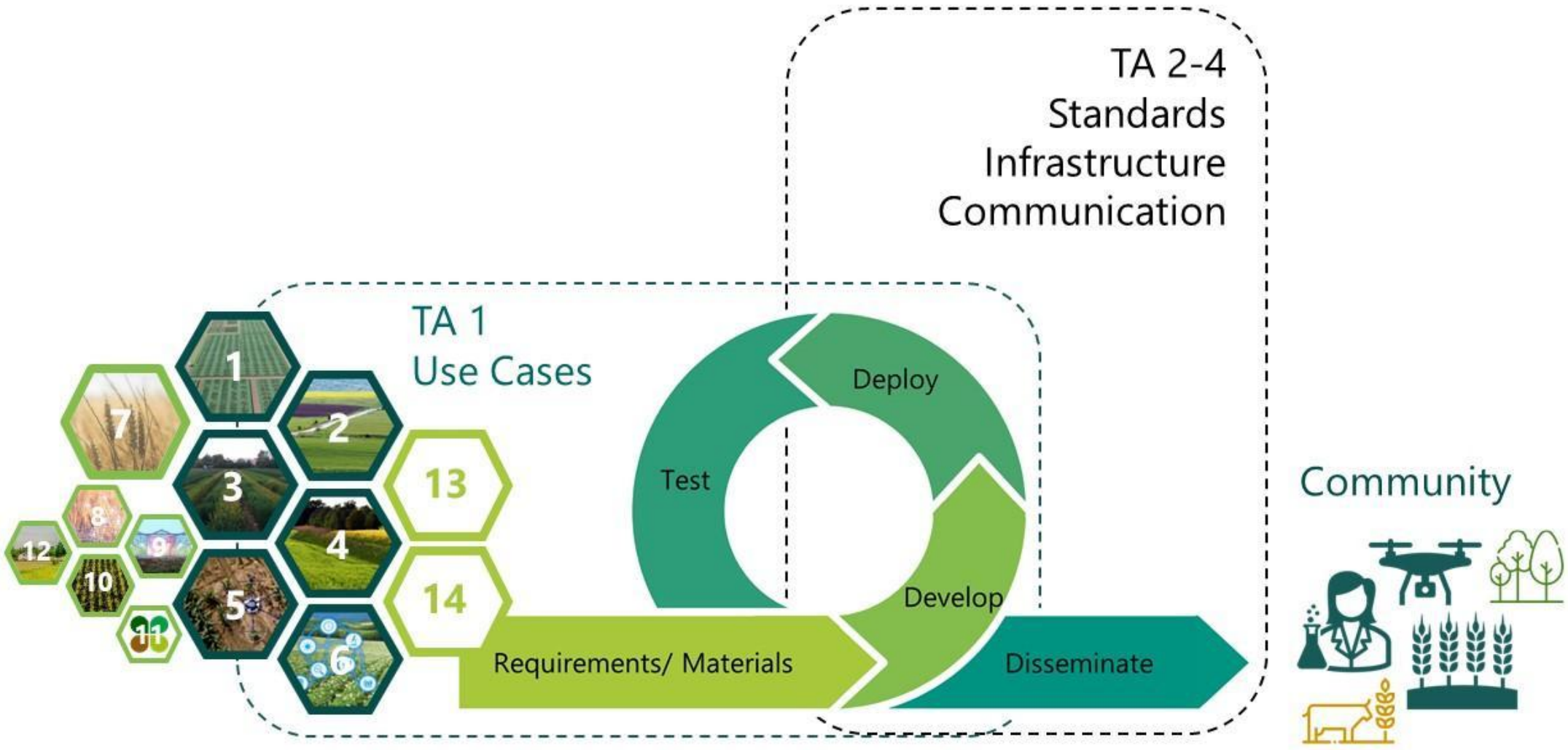


**Figure 6:** The FAIRagro UC cycle, developing RDM solutions together with the community for the community as part of Use Case projects and pilots.

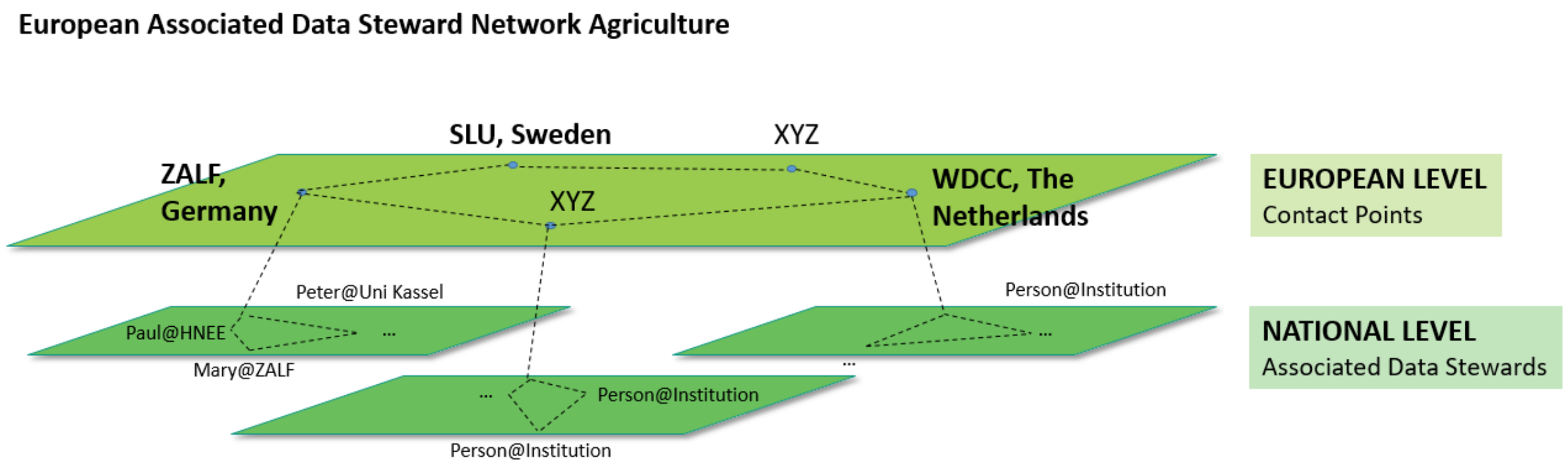


**Figure 7:** Schematic Chart of connecting national and international partners in the European Associated Data Steward Network

**Table 1:** Overview of FAIRagro UC calls and applications, including information on topics and RDM requirements.

| | 1st UC Call (2023/2024) | 2nd UC Call (2025) |
|---|---|---|
| **Number applications** | 12 (3 UC projects, 9 UC pilots) | 16 (11 UC project, 5 UC pilots) |
| **Number funded UCs** | 1 UC (ZALF), 5 UC Pilots (IGZ, ZB MED, Uni Rostock, HU Berlin, IPK) | 2 UCs (HSOS, UFZ) |
| **Evaluation criteria (weight)** | Relevance for FAIRagro (25%) RDM challenge & innovation (25%)<br>Technical feasibility & work plan (25%)<br>Community integration & impact (25%) | Relevance of research & RDM challenge (15%)<br>Development and Implementation of solution (15%)<br>Demonstration of FAIR Practices (15%)<br>Workplan and sustainability (15%)<br>Strategic fit & added value (40%) |
| **Applicant institutions** | ZALF, HNEE, ZEPP, HU Berlin, JKI, IGZ, IPK, Uni Halle (2x), Uni Rostock, ZB MED, Uni Kassel | DBFZ, FBN, FZ Jülich, HNEE, HSOS, HSWT, IGZ, JKI, Thünen Institute (2x), UFZ, ATB, IPK (2x), JLU Gießen |
| **Disciplines addressed** | Crop science, agroforestry, pest & disease monitoring, horticultural science, socio-economic ag research, phenotyping, genomics/genotyping, geospatial data | Plant science, phenotyping/microphenomics, agroforestry, livestock sciences, soil science & digital soil mapping, geophysics, archaeology, socio-economics, water & biodiversity monitoring, remote sensing |
| **Thematic focus** | Climate/extreme events & resilience (ZALF), agroforestry data infrastructure (HNEE), pest monitoring data (ZEPP), horticultural data synchronization (IGZ), AI-supported metadata enrichment (ZB MED), weed & LTE data (Uni Rostock), socio-economic data linkage (HU Berlin), BrAPI integration in phenotyping (IPK), genetic/breeder data FAIRification (Uni Kassel/Halle) | FAIR image datasets & AI workflows (HSOS), geophysical/agricultural data harmonization (UFZ), high-throughput microscopy metadata (IPK), livestock biosample harmonization (FBN), sensor data for animal husbandry (ATB), digital soil mapping quality frameworks (Thünen), IACS livestock/land-use harmonization (Thünen), genebank BrAPI integration (IPK) |
| **RDM challenges** | Fragmented infrastructures, lack of metadata standards (LTE, horticulture, genomics), unclear legal/licensing frameworks, overly ambitious infrastructure-from-scratch approaches (HNEE), limited interoperability, missing repository integration | Metadata harmonization across domains (soil, livestock, geophysics, plant imaging), cross-institutional biosample/data sharing, legal frameworks for sensitive data (livestock, socio-economic), workflow integration into FAIRagro middleware, sustainability of tools beyond pilots |
| **RDM requirements** | Standardized APIs (e.g. BrAPI), harmonized metadata templates (LTE, horticulture, agroforestry, socio-economic), integration with existing RDIs (e!DAL, BonaRes, OpenAgrar), pragmatic legal/ethical guidelines | Interoperable vocabularies & ontologies (MIABIS, BrAPI, soil vocabularies), machine-actionable policies, integration of ARC & RO-Crate for workflows, repository embedding & FAIR Search Hub use, cross-consortia collaboration (NFDI4Earth, NFDI4Objects, NFDI4Biodiversity, DataPLANT) |

# Supplementary Material

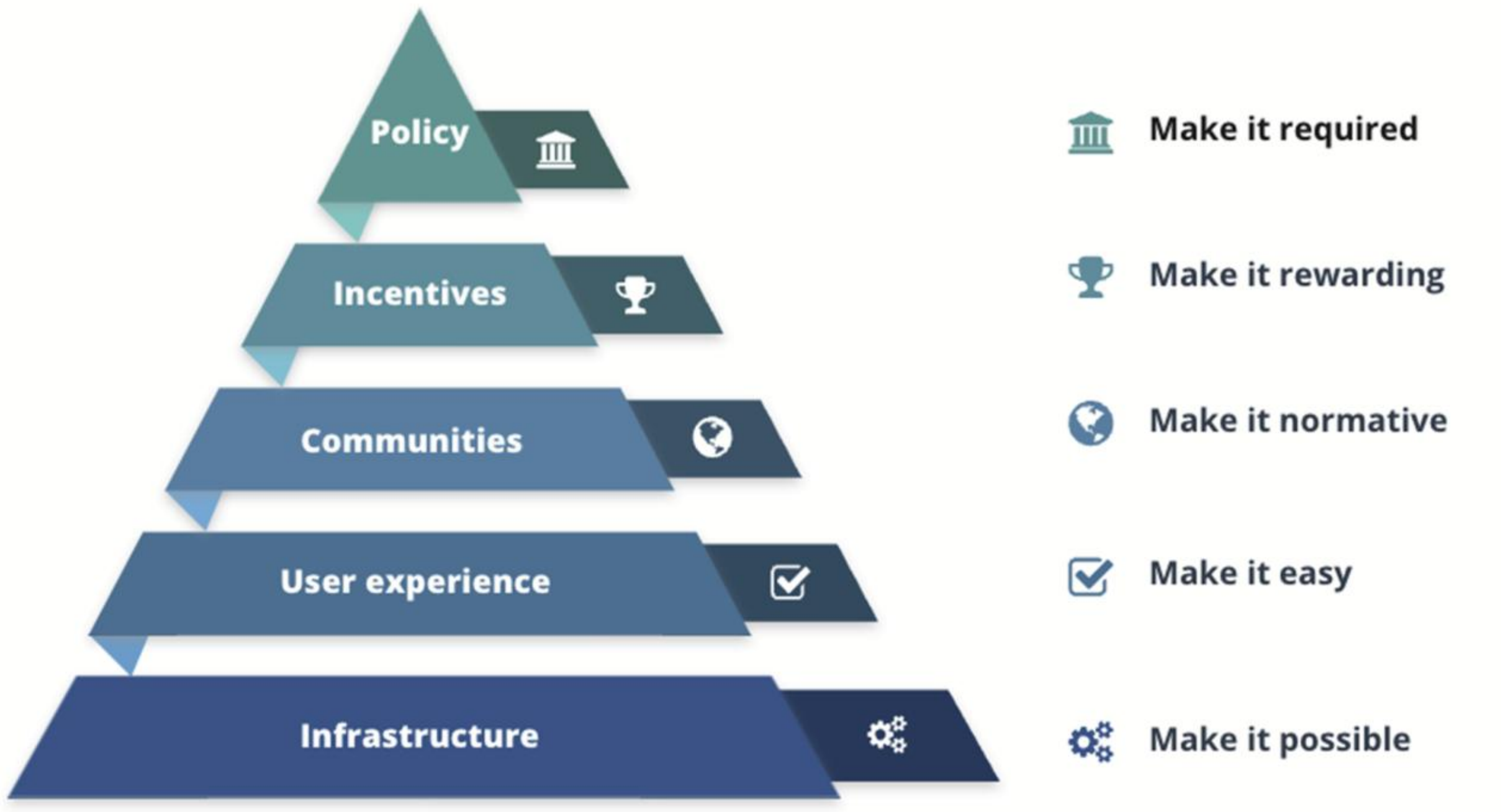


Supplementary Material 1: Figure Nosek's model of culture change from the Center for Open Science blogpost on the strategy plan for sustainable adoption of open behaviors by researchers (Source: Center for Open Science [www.cos.io/blog/continuing-acceleration-new-strategic-plan](www.cos.io/blog/continuing-acceleration-new-strategic-plan) (Creative Commons BY 4.0 International license))

Supplementary Material 2: Summary of Fairagro Community Advisory Board (CAB) Reports

Supplementary Material 3: FAIRagro Interactive Use Case Map and the cross Use Cases - Task Areas working groups
